# Patient satisfaction with infection prevention and control interventions in acute hospitals: a systematic review and meta-analysis

**DOI:** 10.1136/bmjopen-2025-103431

**Published:** 2025-12-14

**Authors:** Mairead Skally, Aoife Kearney, Judith Strawbridge, John Heritage, Cheryl Cox, Kathleen E Bennett, Hilary Humpreys, Fidelma Fitzpatrick

**Affiliations:** 1 Department of Microbiology, Beaumont Hospital, Dublin, Ireland; 2 Department of Clinical Microbiology, Royal College of Surgeons in Ireland, Dublin, Leinster, Ireland; 3 School of Pharmacy and Biomolecular Sciences, Royal College of Surgeons in Ireland, Dublin, Ireland; 4 European Society of Clinical Microbiology and Infectious Diseases (ESCMID) Study Group for Clostridioides difficile, Basel, Switzerland; 5 Patient and Public Involvement Partner, Royal College of Surgeons in Ireland, Dublin, Leinster, Ireland; 6 Data Science Centre, School of Population Health, Royal College of Surgeons in Ireland, Dublin, Ireland

**Keywords:** Infection control, Patient Satisfaction, Systematic Review, Meta-Analysis, Patient Participation, Surveys and Questionnaires

## Abstract

**Abstract:**

**Introduction:**

Infection prevention and control (IPC) interventions are multifactorial and are used to prevent healthcare-associated infections in healthcare facilities. However, patient views and enabling patient and public involvement (PPI) in their development has been minimal.

**Objectives:**

This systematic review aims to identify peer-reviewed publications reporting patient satisfaction outcomes in the context of IPC interventions, to document the methods used to assess patient satisfaction and to conduct a meta-analysis on reported satisfaction outcomes.

**Design:**

Systematic review and meta-analysis following the Joanna Briggs Institute (JBI) methodology and the PRISMA statement, with oversight from a steering group including PPI partners. Studies in peer-reviewed journals were included based on eligibility criteria.

**Data sources:**

MEDLINE, Scopus, Web of Science, EMBASE, Cochrane Library, CINAHL and PsycINFO were searched in June 2024.

**Eligibility criteria:**

Included studies investigated satisfaction among hospitalised patients in acute care settings following IPC measures, including isolation, cohorting, screening, hand hygiene, antimicrobial stewardship, patient flagging, education, personal protective equipment use, visiting restrictions and treatment delays

**Data extraction and synthesis:**

Titles and abstracts were screened independently by two reviewers; disagreements were resolved by a third. Study quality was assessed using the JBI manual for evidence synthesis. A meta-analysis was conducted where four or more studies used comparable designs and methods within the same areas of IPC, with heterogeneity evaluated using Cochran’s Q statistic and I^2^ and pooled estimates calculated with 95% CIs using the Wilson (score) method.

**Results:**

Twenty-nine studies were identified. Among IPC measures, isolation precautions were the most commonly reported intervention (11 studies, 38%). The Likert scale was the predominant assessment method (13 studies, 45%). Patient satisfaction with IPC interventions ranged from 58.3% to 97.2%. Meta-analysis of four studies using the Hospital Consumer Assessment of Healthcare Providers and Systems survey showed substantial heterogeneity (I^2^, 55%, p=0.08) and a pooled patient satisfaction level of 69% (95% CI 63.6% to 74.4%) for isolation precautions.

**Conclusion:**

Sixty-nine percent of isolated patients reported satisfaction with their care. Patient satisfaction with IPC interventions varies widely, highlighting limitations in current measurement approaches. Strengthening PPI in the design and evaluation of satisfaction measures is essential to capture meaningful data and improvements in IPC programmes.

**PROSPERO registration number:**

IS 2024 CRD42024558385.

**STRENGTHS AND LIMITATIONS OF THIS STUDY**

- This systematic review aims to evaluate and elucidate patient satisfaction in relation to infection prevention and control (IPC) interventions, considering it as a distinct indicator of healthcare quality; specifically, to assess the degree to which patient expectations are met and the methods used to measure and quantify satisfaction across studies.
- The results demonstrate marked heterogeneity in both the types of IPC interventions reported and the methods used to measure patient satisfaction, while inadequate reporting of comparator groups limited the meta-analysis to pooled proportions within IPC cohorts.
- The studies focused on meticillin-resistant *Staphylococcus aureus* and COVID-19, with no studies involving patients exposed to other common infections, such as *Clostridioides difficile* infection or norovirus.
- This systematic review demonstrates the feasibility of using questionnaire-based surveys to assess whether patients’ expectations of their care were met and to highlight areas requiring improvement; however, a consensus on a standardised approach to measuring patient satisfaction in the context of IPC is needed.

##  Introduction

Infection prevention and control (IPC) interventions in healthcare facilities aim to prevent healthcare-associated infections (HAIs) among patients and staff.^1^,^3^ IPC measures are multifactorial and include horizontal interventions like hand hygiene and respiratory etiquette and vertical measures like isolation precautions and patient cohorting. These interventions are widely recommended by national and international bodies,^2 4^ based mainly on scientific knowledge, with minimal patient and public involvement (PPI) in their development.

PPI can support quality improvement of IPC services.^5^ PPI enables service-users to apply their priorities to healthcare evaluation, development, organisation and delivery.^6^ While the importance of PPI for IPC-related policy and guideline development is central to implementing local and global measures (eg, restricted movement during the COVID-19 pandemic),^7 8^ there remains no single strategy to indicate how best to engage and embed PPI. Systematic reviews in the area show low frequency of PPI reporting in patient safety research.^9 10^

Assessing whether patient expectations are met is key to capturing the patient voice. Two commonly used measures are ‘patient experience’ and ‘patient satisfaction’. Although often used interchangeably, they represent distinct concepts. Patient experience reflects patients’ views of care relative to their expectations, while patient satisfaction measures whether or not those expectations were met. Qualitative studies of patient experience may provide deeper insights into perceptions of care, whereas quantitative satisfaction measures provide ratings that allow for systematic synthesis where methods and measures are comparable. Patient satisfaction is crucial for health service quality.^11^,^14^ As different services are offered within healthcare facilities, overall patient satisfaction and transaction-specific satisfaction (ie, with IPC services) should be differentiated.^15^

So, how satisfied are patients with IPC services? Previous systematic reviews have focused either on ‘patient satisfaction’ or on only one IPC measure. To date, no systematic review has addressed patient satisfaction with IPC interventions, whether assessed directly or as part of broader factors such as general hygiene.^1216^,^18^ As such, it is not known whether patients’ expectations of IPC measures are being met. This systematic review aimed to identify peer-reviewed publications reporting patient satisfaction outcomes in the context of implemented IPC interventions, whether satisfaction was the primary focus or one of several factors examined, to document the methods used to quantify patient satisfaction and to conduct a meta-analysis of reported satisfaction levels associated with specific IPC interventions where feasible.

## Methods

The concept and design of this systematic review was overseen by a steering group that included PPI partners. This systematic review (PROSPERO protocol registration number: IS 2024 CRD42024558385) was conducted in accordance with the Joanna Briggs Institute (JBI) methodology for systematic reviews of aetiology and risk.^19^ The Preferred Reporting Items for Systematic Reviews and Meta-Analysis statement was followed.^20^ The full study protocol has been published previously.^21^ The only variation from the published protocol is the use of the appropriate JBI checklist for quality assessment due to diversity of included studies.

### Patient and public involvement

Two PPI partners (JH and CC) were members of the project’s steering group. The study concept and design were developed under the guidance of this group. The steering group noted that while hospitals routinely collect patient-experience data, feedback specific to IPC interventions is rarely documented; this observation informed the review question. The PPI partners contributed throughout the study, including protocol development, methodological review and interpretation of findings.

### Eligibility criteria

Studies in peer-reviewed journals meeting the search criteria were assessed for inclusion. A population, exposure, outcomes (PEO) framework was applied to guide study selection. The population of this review comprised hospitalised patients in acute care hospitals. Exposures included a range of IPC interventions, either as a broad category or specific strategies, such as isolation precautions, patient cohorting, screening for multidrug-resistant organisms (MDROs), hand hygiene, antimicrobial stewardship, MDRO patient flagging, IPC-related education and communication, the use of personal protective equipment (PPE) by staff, visitors or patients, restricted visitor policies and delays in transfer, discharge or procedures. The outcome of interest was patient satisfaction following exposure to IPC interventions, measured using self-administered surveys or interviews. Where available, comparator data (ie, either usual care without the specified IPC intervention(s) or alternative IPC strategies) were noted but were not a requirement for inclusion. No language or time restrictions were applied. Studies of non-admitted patients, patients aged <18 years or without a quantifiable measure of patient satisfaction were excluded. Case reports, case series, letters, editorials, meta-analyses, commentaries, review articles and conference abstracts were also excluded. Reference lists of systematic reviews identified during the literature search were searched for eligible studies.

### Search terms

Search strategy information is provided in online supplemental material 1. Search strings focused on combining terms for ‘infection prevention and control’ and ‘patient satisfaction’ to ensure all results were captured. To account for the wide range of recommended IPC measures,^2 4^ search terms were intentionally kept broad and generic, for example, “isolation precautions”, “hand hygiene”, so as to exhaustively search the literature.

### Information sources

Searches were conducted across a broad range of databases. MEDLINE, Scopus, Web of Science, EMBASE, Cochrane Library, Cumulative Index to Nursing and Allied Health Literature and PsycINFO databases were searched electronically in June 2024.

### Selection process

Studies were imported to Rayyan Systematic Review software, and duplicates were removed.^22^ Titles and abstracts were screened and assessed for eligibility, as outlined above, independently by two reviewers, MS and AK. Studies reporting on patient satisfaction and general hygiene were assessed for eligibility as the cleanliness of the hospital environment is recognised as a key component of IPC in healthcare settings.^2 4^ Studies were eligible if they reported patient satisfaction in the context of implemented IPC interventions, or in relation to IPC-related outcomes (eg, surgical site infection (SSI)), given the direct association between these outcomes and IPC effectiveness. Conflicts were resolved by HH and final numbers of included studies displayed as per the Preferred Reporting Items for Systematic Reviews and Meta-Analysis (PRISMA) format (table 1).^20^

**Table 1:** Determining patient satisfaction among included studies based on how this was measured and reported

Measurement scale/method	Criteria used in this review to determine patient satisfaction
5-point Likert scale	Scoring either 4 or 5 on 5-point scale
Hospital Consumer Assessment of Healthcare Providers and Systems	‘Top box’ scores, that is, responding ‘always’ or ‘yes’ when asked if they were satisfied with their care OR Scoring either 9 or 10 on 10-point Likert scale
Other	If overall scores with a measure were presented, the number of patients was calculated by working backwards from the satisfaction calculation formula, where possible. These calculated fields are marked as such on the data extraction tables2 3.

**Table 2:** Summary of papers identified reporting patient satisfaction with infection prevention and control (IPC) measures

Year published	Number of studies (n=29)	Number of studies by scale used			
		Likert scale	HCAHPS	Not reported	Other
pre-2010	4	1	1	1	1
2010–2015	10	4	5		1
2016–2020	10	5	2	2	1
2021–June 2024	5	3			2
Country/region					
European based					
France	3	3			
UK	2	1			1
Germany	1				1
Italy	1	1			
Spain	1	1			
USA	10	2	8		
Pakistan	2	1		1	
China	2			1	1
Iran	2	2			
Canada	1			1	
Ethiopia	1	1			
Hong Kong	1				1
Singapore and Philippines	1	1			
Turkey	1	1			
**IPC measures studied**					
Isolation precautions	11	3	5	1	2
General hygiene	8	5	1	1	1
Surgical site infection	3	2	1		
COVID-19	2	1	1		
Intravascular devices	2				2
Other	3	2		1	
**Study design**					
Case control	3	1	1		1
Case series	2	1		1	
Cohort	5	1	3	1	
Cross sectional	10	7	1	1	1
Quasi experimental	6	2	3	1	
RCT	3	2		1	
**Mode of data capture**					
Interview	3	1	1	1	
Questionnaire	17	11		4	2
Survey	9	2	7		

HCAHPS, Hospital Consumer Assessment of Healthcare Providers and Systems; RCT, randomised control trial.

**Table 3:** Studies where infection prevention and control (IPC) measures and patient satisfaction were reported by IPC type, data collection method, scale and non-IPC cohort

Author	Country/region	Period	Scale used	Points on scale	Description of points on scale	Primary outcome reported no. of satisfied patients/no. surveyed (%)	Study design	Overall quality of study
**Isolation precautions studies**								
Chittick *et al*^31^	USA	01 January 2014–31 December 2014	Likert	5	1=disagree strongly, 5=agree strongly	Overall satisfaction: 206/249 (82%)	Quasi-experimental	Moderate
Gasink *et al*^26^	USA	07 August and 25 August 2006	HCAHPS	Scale of 0–10	11	Overall satisfaction: median rating of 9 (IQR, 8–10)	Cross-sectional	High
Gaube *et al*^34^	Germany	June 2021–February 2022	PPE15	Dichotomous (0=no issue, 1=issue).	15-item picker patient experience	Overall dissatisfaction: mean score 6.20, SD 4.08	Case-control	High
Guilley-Lerondeau *et al*^32^	France	March–July 2012	Likert	4	Very dissatisfied, dissatisfied, satisfied, very satisfied	Overall satisfaction: 25/30 (83.3%)	Cohort	High
Lau *et al*^35^	Canada	10 October 2013–2 November 2014	Not reported	Not reported	Not reported	Mean score for overall satisfaction: 8.39, SD 1.68	Cohort	High
Livorsi *et al*^27^	USA	1 January 2012–31 May 2012 and 1 June 2012–31 March 2013	HCAHPS	0–10, where 0 is worst and 10 is best	11	Overall satisfaction: 44/68 (64.7%)	Case-control	High
Lupión-Mendoza *et al*^33^	Spain	2011–2012	Likert	Not reported	Not reported	Overall satisfaction with care received from professionals: 67/72 (93.1%)	Case-control	High
Mehrotra *et al*^28^	USA	11 January 2010–17 November 2010	HCAHPS	Not reported	Not reported	Overall satisfaction: 22/37 (58.3%)	Cohort	High
Rees *et al*^36^	UK	Not reported	Not reported	11	Not reported	Mean score for overall satisfaction: 7.62, SD 2.06	Case series	High
Siddiqui *et al*^29^	USA	July 2011 and July 2016	HCAHPS	Not reported	Not reported	Overall satisfaction: 1302/1784 (73%)	Quasi-experimental	High
Vinski *et al*^30^	USA	1 January 2010 and 30 September 2010.	HCAHPS	Scale of 1–10	10	Overall satisfaction:133/195 (68%)	Quasi-experimental	High
**General hygiene studies**								
Erden and Emirzeoglu^37^	Turkey	July 2018–December 2019	Likert	5	1=never, 2=very poor, 3=poor, 4=often, 5=always	Mean score for overall satisfaction: 3.25, SD 1.05	Cross-sectional	High
Formal and Riddell^44^	USA	2011–2012	HCAHPS	Not reported	Not reported	Overall satisfaction 91%	Quasi-experimental	High
Hussain *et al*^38^	Pakistan	December 2010–February 2012	Likert	5	1=disagree strongly, 5=agree strongly	Satisfaction with general hygiene of ward: 453/710 (63.8%)	Cross-sectional	High
Lucadamo *et al*^39^	Italy	First semester 2018	Likert	7	1=minimum level of satisfaction, 7=full satisfaction.	Not reported	Cross-sectional	High
Tasneem *et al*^43^	Pakistan	June 2010	Not reported	3	Satisfied, dissatisfied, undecided	Satisfaction with general cleanliness: 173/190 (91.1%)	Cross-sectional	High
Woldeyohanes *et al*^40^	Ethiopia	08 May–02 June 2011	Likert	5	1 very satisfied to 5=very dissatisfied	Overall satisfaction: 117/189 (61.9%)	Cross-sectional	High
Wong *et al*^42^	Hong Kong	June–October 2010	HKIEQ	5	Excellent/very good, good, fair, poor, very poor	Overall satisfaction: 4029/5030 (80.1%)	Cross-sectional	High
Ziapour *et al*^41^	Iran	January–March 2014	Likert	5	1 for very bad responses, 5 for very good	The patients’ total satisfaction with medical services was 3.49 out of 5.	Cross-sectional	High
**Surgical site infection-related studies**								
Boulet *et al*^45^	France	October 2018–January 2019	Likert	4	Very satisfied/rather satisfied/rather dissatisfied/very dissatisfied	Satisfaction with preoperative shower process: 363/430 (84.4%)	Cross-sectional	High
Day *et al*^47^	USA	2010–2011	HCAHPS	Scale of 1–10	Normalised to 100-point scale	Overall satisfaction score: 94.8, n=155†	Cohort	High
Merle *et al*^46^	France	October/November 2005 and April–August 2006	Likert	4	Very satisfied, rather satisfied, rather not satisfied and not at all satisfied use	Satisfaction with information provided regarding SSI: 58/87 (67%)	RCT	High
**COVID-19 studies**								
Farajzadeh *et al*^48^	Iran	2020	Likert	5	0=completely dissatisfied, to 4 completely satisfied	Overall satisfaction: 63/73 (86%)‡	Cross-sectional	High
Zeh *et al*^49^	USA	2 February–16 April 2020	HCAHPS	Not reported	Not reported	Overall satisfaction: 35/59 (66.0%)	Cohort	High
**Intravascular device studies**								
Prasad *et al*^51^	Singapore & Philippines	November 2018–February 2019	Likert	5	1 very dissatisfied, 5 very satisfied	Overall satisfaction with catheterisation: 471/544 (86.6%)§	Quasi-experimental	Moderate
Sun *et al*^50^	China	June 2017–May 2019	Not reported	3-point scale, study specific	Excellent: >90%, good: 70%–89%, poor: <70%	Overall satisfaction: 103/106 (97.2%)	RCT	High
**Other studies**								
Bellamy^52^	UK	18-month period year not specified	Likert	5	Not reported	Satisfaction with IPC measures used when caring for patients with MRSA: 36/46 (78.2%)¶	Case series	Moderate
Schulte *et al*^53^	USA	Not reported	Likert	5	1 representing ‘not satisfied at all’ and 5 representing ‘extremely satisfied’	Satisfaction with intervention for smoking cessation: 13/17 (76.5%)¶	RCT	Moderate
Liu and Sun^54^	China	April 2016–2017	Not reported	Not reported	Not reported	Hand hygiene with the introduction of group: 57/60 (95.6%)	Quasi-experimental	High

* Calculated by averaging results across sites.

† Scores were normalised to a 100-point scale.

‡ Translated version of paper used.

§ Domains surveyed: infection management, professionalism of staff, effectiveness of communication, physical comfort, pain management.

¶ Calculated using an average satisfaction score for ‘Satisfied’ and ‘Very satisfied’ categories and calculation the overall score by summing individual question scores.

HCAHPS, Hospital Consumer Assessment of Healthcare Providers and System; HKIEQ, Hong Kong Inpatient Experience Questionnaire; MRSA, Meticillin-resistant *Staphylococcus aureus*; PPE15, Picker Patient Experience Questionnaire; RCT, randomised control trial; SSI, Surgical site infections.

### Data extraction

MS completed full-text review and data extraction of studies meeting inclusion criteria. AK completed this process in duplicate for 20% of included studies. Discrepancies were resolved through discussion, with arbitration by a third reviewer (HH) if needed. No a priori discrepancy threshold was set as this process served as a quality check to ensure consistency prior to single data extraction. Data were extracted into a Microsoft Excel file and included title, authors, year, journal, study design, intervention, data capture method and reported satisfaction scores. Numbers of satisfied patients in both IPC and non-IPC cohorts were extracted, where possible.

### Risk of bias in individual studies

The study design of each study was determined and a critical appraisal of the paper performed by MS using the appropriate JBI checklist according to their manual for evidence synthesis.^23^ AK completed this process in duplicate for the 20% of included studies. All included studies, regardless of methodological quality assessment, underwent data extraction.

### Data synthesis

Study characteristics were summarised by author, year, country, number of facilities included and study period. Outcome variables extracted included: patient groups observed, design of method and scale used to measure satisfaction, satisfaction criteria, primary outcome reported, number of IPC patients included and the proportion meeting satisfaction criteria. For each study, the number of satisfied patients was extracted according to authors’ specified definitions and thresholds with no additional reclassification applied. The number of patients in non-IPC comparator groups, if reported, as well as the number of those who were satisfied, the study design and the overall quality of study were also extracted. Due to inconsistent reporting of comparator data across studies, a quantitative synthesis of absolute or relative differences between IPC and non-IPC cohorts was not feasible. For studies where comparator data were available, the results have been presented within the main dataset.

### Statistical analysis

A random effects meta-analysis of pooled proportions was conducted where four or more studies used comparable designs and measurement methods within the same areas of IPC. Proportions were pooled using Stata’s meta routine, using untransformed proportions,^24^ and a random effects model applied using Restricted Maximum Likelihood. Cochran’s Q statistic and I^2^ were used for heterogeneity. A p value <0.10 for the Cochran’s Q test or I ^2^>50% indicate heterogeneity between studies.^25^ All analyses were performed using Stata V.18.5.

## Results

**Figure 1:**
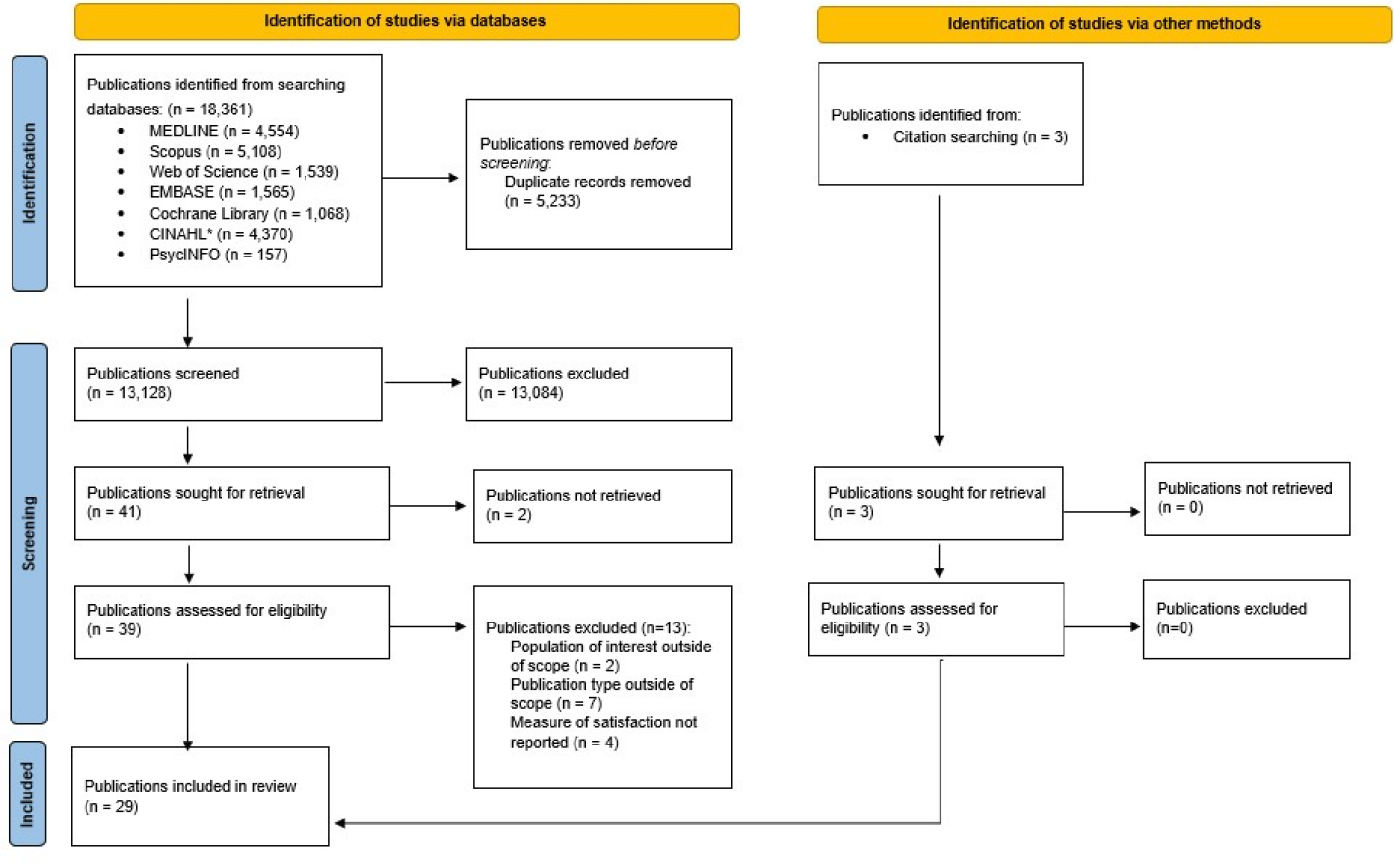
Preferred Reporting Items for Systematic Reviews and Meta-Analysis flow diagram of included studies. Records identified, screened and excluded are shown at each stage.

The literature search conducted in June 2024 identified 18 361 publications (figure 1). Following duplicate removal (n=5233), abstract/title screening (n=13 128), assessment of full-text eligibility (n=41) and a search of citations, 29 studies were included. Defining ‘satisfied’ was dependent on the data collection method used by the authors and is shown in table 1. Study designs included case-control (n=3), case series (n=2), cohort (n=5), cross-sectional (n=10), quasi-experimental (n=6) and randomised control trials (n=3).

Isolation precautions were the most common IPC measure reported (n=11/29, 38%) followed by general hygiene (n=8/29; 28%) and SSI prevention (n=3/29, 10%). The Likert scale was the most reported assessment method (n=13/29) (table 2). Most studies from the USA reported findings from the country’s national Hospital Consumer Assessment of Healthcare Providers and System (HCAHPS) survey (n=8/29). Table 3 provides an overview of each study. Full details are provided in online supplemental material 2.

### Risk of bias and overall quality of evidence

Study design was determined and the appropriate JBI checklist completed for each study. Individual study checklists are provided in online supplemental material 3. Most of the included studies were considered high quality (n=25, 86%) (table 2). Those of moderate quality (n=4, 14%) reported information relevant to the subject area and were included to ensure representation of a comprehensive overview of the literature landscape.

### Isolation precautions

**Figure 2:**
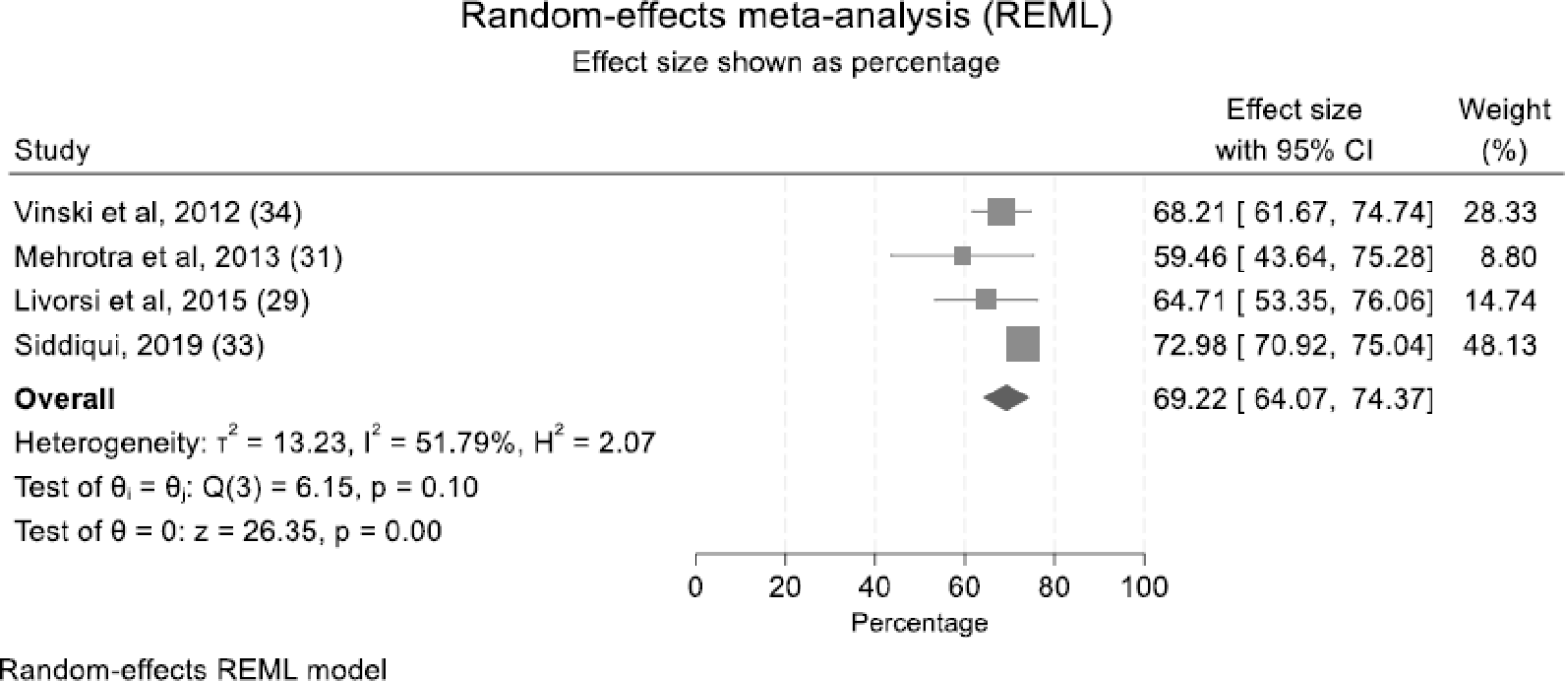
Forest plot of proportions of satisfied patients undergoing isolation precautions. Each row represents one study in the meta-analysis, with proportion of satisfied patients under isolation precautions (box) plotted with 95% CI (horizontal line). The diamond represents the pooled proportion of satisfied patients under isolation and estimated 95% CI.

Eleven studies (published 2000–2023) reported satisfaction with isolation precautions, 10 high quality and one moderate. Most (n=8/11) reported overall hospital satisfaction among isolated patients, while three examined specific populations or satisfaction domains. Five studies (sample sizes ranging 37–1748) used the HACHPS survey method.^26^,^30^ Gasink *et al* performed a cross-sectional study in a 1070-bedded tertiary care centre, reporting a median of nine (IQR: 8–10) for isolated versus eight (IQR 7–10) among non-isolated patients.^26^ The other four studies using the HCAHPS method reported ‘top box’ responses among isolated patients (range 58.3–73%).^27^,^30^ The lowest ‘top box’ proportion (58.3%; vs 70% among non-isolated patients) was reported by Mehrotra *et al*^28^ and the highest 68% among versus 74% for 7806 non-isolated patients by Vinski *et al*. A random-effects meta-analysis of pooled proportions of these four studies showed some heterogeneity (l^2^=55.7%, p=0.08).^27^,^30^ The pooled level of patient satisfaction of patients under isolation precautions was 0.69 (95% CI 0.64 to 0.74), indicating 31% of individuals under isolation precautions were not satisfied with their care (figure 2).

Three studies conducted in the USA, France and Spain reported on the satisfaction of isolated patients using the Likert scale.^31^,^33^ Chittick *et al* (moderate quality) reported 82% overall hospital satisfaction using the Likert scale noting HCAHPS was unsuitable as it is ‘retrospective’ with limited scope.^31^ Guilley-Lerondeau *et al* (high quality) found 83.3% satisfaction in isolated patients versus 100% in non-isolated patients using a four-point scale.^32^ Lupión-Mendoza *et al* (high quality) reported 93.1% satisfaction with ‘care received from professionals’ in Spain, though overall satisfaction of the hospital and scale details were not provided.^33^

Gaube *et al* (high quality) evaluated patient dissatisfaction associated with isolation precautions using the Picker Patient Experience Questionnaire.^34^ This employs a dichotomous scoring method to generate a ‘problem score’, where responses are coded as either 0 or 1 and subsequently summed. The higher the score, the greater the patient dissatisfaction. Among isolated patients (n=118) the mean problem score was 6.20 (SD 4.08), compared with a mean score of 4.85 (SD 3.11) reported by non-isolated patients.

Two studies did not specify data collection methods.^35 36^ Rees *et al* (high quality) reported a mean satisfaction score of 7.62 (SD 2.06) on a 0–10 scale among isolated patients, with no control group. Lau *et al* (high quality) reported mean discharge satisfaction scores of 8.39 (SD 1.68) for isolated patients and 8.50 (SD 1.87) for non-isolated patients. No details on scales used were given.^35^

### General hygiene

Eight high-quality studies (published 2010–2020) reported patient satisfaction with general hospital hygiene as part of overall experience. Five studies from Ethiopia, Pakistan, Iran, Turkey and Italy used Likert scales.^37^,^41^ Four studies used five point scales. Woldeyohanes *et al* reported 76.8% satisfaction with ward hygiene,^40^ Hussain *et al* reported general hygiene at 63.8% with general hygiene.^38^ Ziapour *et al* reported mean satisfaction with general hygiene 3.26/5 (SD not reported)^41^ and Erden and Emirzeoglu reported mean satisfaction for room cleanliness of 3.58 (SD 0.78).^37^ Lucadamo *et al* used a seven-point scale to construct a statistical model for evaluating patient satisfaction,^39^ but no comparable data were extractable.

Wong *et al* found 98.6% of patients reported the hospital as ‘very clean’ or ‘fairly clean’ using the Hong Kong Inpatient Experience Questionnaire, a five-point scale’^42^ .

Tasneem *et al* found 91.3% of patients satisfied overall with the general cleanliness of the hospital.^43^ Hussain *et al*, using a Likert scale, found 63.8% were satisfied with ward hygiene.^38^ Fornwalt and Riddell used HCAHPS data to assess changes in patient satisfaction with general hygiene after introducing ultraviolet environmental cleaning.^44^ Satisfaction increased from 79.6% to 91%; however, no denominator data were provided.

### Surgical site infection prevention

Three studies, of high quality, reported patient satisfaction with SSI, two on prevention^45 46^ and one with postoperative infection care.^47^ Both studies investigating SSI prevention used a four-point scale. Merle *et al* found satisfaction with information received about SSI prevention with leaflets was 66.7% compared with 43% without leaflets.^46^ Boulet *et al* found patient satisfaction with preoperative showers^45^ to be 84.4%. Neither study reported results from a comparator group. Day *et al*, using HCAHPS data and scores that were normalised to a 100-point scale, reported a satisfaction score of 94.8 among patients with an SSI, compared with 94.0 of patients without an SSI.^47^

### COVID-19

Two high-quality studies conducted in 2020 reported patient satisfaction with the hospitals’ overall COVID-19 measures.^48 49^ Farajzadeh *et al* used a five-point Likert scale to report satisfaction of 86% among COVID-19 patients post discharge.^48^ Zeh *et al* compared the satisfaction of surgical patients with and without visitor restrictions, during the initial period of the COVID-19 pandemic.^49^ Fewer patients reported ‘complete satisfaction’ (66%) in the restricted visiting group, compared with 80.7% without restrictions.

### Intravascular device infection prevention

Two studies investigated the satisfaction of patients with intravascular devices (IVDs). Sun *et al* (high quality) evaluated the impact of care bundles for preventing central venous catheter-associated infections on patient satisfaction.^50^ No data collection method was described. Satisfaction of patients with the bundles was 97.2% compared with 85.8% of patients without the bundle. Omkar Prasad *et al* (moderate quality) used a five-point Likert scale to examine vascular access management and patient satisfaction with infection management, professionalism of healthcare workers, communication, physical comfort and pain management,^51^ with 86.6% of patients ‘satisfied’ with the different domains.

### Other studies

There were three additional studies identified covering elements of IPC not already discussed.^52^,^54^ Each reported on patient satisfaction with a specific area of IPC, and none of the three reported on overall hospital satisfaction.

The study by Bellamy *et al* (moderate quality) investigated patients with methicillin-resistant *Staphylococcus aureus* (MRSA) satisfaction with service and treatment of 78.2% using a five-point Likert scale.^52^ Satisfaction for patients receiving usual care was not reported. Schulte *et al* (moderate quality) studied patient satisfaction using a five-point Likert scale with a smoking cessation initiative, noting that smoking increases HAI risk.^53^ Satisfaction with the intervention was 76.5%.

Liu and Sun (high quality) introduced group nursing as an intervention to improve hand hygiene.^54^ How satisfaction was measured was not reported. Of patients with group nursing, satisfaction was 95.6% compared to 83.8% without.

## Discussion

This systematic review identified 29 studies reporting patient satisfaction outcomes in the context of IPC interventions. The findings revealed considerable heterogeneity, both in the types of IPC interventions studied and in the methodologies used to assess satisfaction, including survey instruments and rating scales used. Meta-analysis was possible for only four studies that used the HCAHPS survey to assess patients’ views during isolation. These studies reported a satisfaction level of 69% among isolated patients.

Key performance indicators (KPIs) are metrics accepted as an international standard for performance monitoring and benchmarking of healthcare facilities by national agencies. KPIs are important for effective management and the prevention of HAI and for identification of areas for improvement.^55^ While such measures are crude, they are consistent and routinely interrogated.^56^ There are several studies reporting on the patient experience and their views on HAI.^57^,^59^ As this is a subjective measure, these studies are limited in terms of scale and scope. Patient satisfaction offers a more systematic, metric type approach to capturing patient feedback. However, the findings of this review show disparity even when the same measurement methods have been used, that is, Likert scale (ranging 4–7 points) and HCAHPS (reported differently). While most studies used HCAHPS, limitations were noted including the retrospective nature of such surveys as well as subject material being nonspecific to IPC.

Our findings highlight that many studies relied on generic patient satisfaction tools that may not adequately capture perceptions of IPC-specific practices. There is a need to develop tailored tools that include domains such as hand hygiene, isolation procedures and communication regarding infection risk. Ideally, these tools should be co-designed with patients and members of the public to ensure greater relevance and responsiveness to their concerns. Greater standardisation of IPC-specific satisfaction measures would also facilitate comparability across studies and healthcare settings. Additionally, the review highlights the importance of PPI in the development of tailored tools to measure patient satisfaction with IPC interventions. Future surveys should be designed with meaningful input from patients to ensure that the data captured reflects their experiences and priorities, thereby enabling actionable conclusions. Future studies should focus on issues that concern patients, and not only on those perceived important by IPC teams. Some studies may need to address general IPC concerns, for example, hospital hygiene, hand hygiene and PPE use. However, others should target specific issues, infections or settings, such as SSI, CDI and care in oncology/haematology units.

The quality of the included studies was mostly high (86%). While the presence of moderate/low-quality studies introduces potential risk of bias in results and findings, this did not affect the findings of the meta-analysis, as these studies were excluded from the pooled analyses. As most studies reported only crude satisfaction estimates of patients’ satisfaction, direct comparisons between isolated and non-isolated patient groups were not conducted. Any such comparative analysis would need adjustment for potential confounders such as illness severity, length of hospital stay and other relevant clinical factors.

Isolation precautions, the focus of most of the studies included, are widely recommended for the prevention of HAI and used in conjunction with standard precautions.^60^ Though widely used, it has been reported that isolation has negative consequences for segregated patients, including increased anxiety and psychological suffering.^61^ It is therefore unsurprising that isolation precautions are the most studied element of IPC and patient satisfaction. Meta-analysis of four studies with suitable findings showed that while 69% of patients being isolated were satisfied with their hospital stay, 31% of patients’ expectations with IPC interventions were not met. Patient satisfaction with hospital inpatient care is typically assessed using standardised national surveys, with satisfaction targets for positive overall ratings often set at or above 80%. For example, Ireland’s 2024 national inpatient survey reported that 85% of respondents rated their experience as ‘good’ or ‘very good,’ consistent with international benchmarks.^62^ Likewise, top-performing hospitals in the USA consistently achieve patient satisfaction rates in the 80–85% range as measured by the HCAHPS survey.^63^

General hygiene was the second-most identified area of IPC and patient satisfaction. Though general hygiene is a core principle of good IPC, most of the included studies did not evaluate it in an IPC context. Instead, these studies were generic patient satisfaction type studies, where general hygiene was reported as a subtheme of overall care. Such an approach may bias patients’ views. Patient responses are influenced by context and question framing. The same individual may respond differently to a ‘hospital hygiene’ item in a general experience survey compared with an IPC-specific survey administered during isolation, potentially altering satisfaction estimates independent of actual experience. The healthcare environment is dynamic and complex, especially for patients on their healthcare journey, with numerous interactions across many disciplines. A systems approach in healthcare research involves understanding and improving the complex interactions within healthcare systems to enhance patient outcomes, efficiency, and overall health. It takes a more holistic perspective of the complexity of social processes in the healthcare environment, enabling the study of different system components and their relationships within a wider environment.^64^ Given the breadth and diversity of IPC measures, a systems approach may be useful when developing methods for measuring patient satisfaction and IPC intervention.

Several included studies assessed patient satisfaction in the context of SSI prevention. While SSI represents an outcome rather than a direct IPC intervention, it is a core endpoint of IPC programmes and hence provides indirect insight into patient perceptions of IPC effectiveness, though the findings should be interpreted within this context. The review also found limited reporting on specific topics such as SSI, hand hygiene and IVD care. Despite the recent COVID-19 pandemic, only two studies reported patient satisfaction in that context. The pathogens specifically studied were limited to MRSA and COVID-19. No studies investigated patient experiences of other common causes of acute illness, such as *Clostridioides difficile* or norovirus.

Strengths of this study include the use of a rigorous methodology, adherence to the JBI and PRISMA guidelines and a thorough critical appraisal of included studies. To minimise the risk of missing relevant publications, broad search terms were applied across multiple databases without language restrictions. However, as data extraction and risk of bias assessment were not conducted in full duplicate for all studies, a small potential for error or bias should be considered when interpreting the findings. Additionally, the scope of meta-analysis was limited by the heterogeneity in both IPC interventions reported and patient satisfaction measurement methods. Most studies did not report satisfaction outcomes for non-IPC comparator groups in a manner that allowed for the calculation of absolute or relative differences. As a result, the analysis focused on pooled satisfaction proportions within IPC cohorts rather than comparative effect estimates. The overall quality of 14% of studies was rated as moderate; however, these results were excluded from pooled analysis.

Further research should be extended to include the perspectives of children and carers to provide a more comprehensive understanding and overview of patients experience across diverse populations and healthcare settings.

Surveys are the most feasible, practical method of assessing patient satisfaction. While questionnaire surveys lack detail compared with qualitatively assessing patient experience, they provide a consistent method of data capture from large numbers of patients and allow for systematic synthesis of findings where methods and measures are comparable. This empowers patients to provide an assessment of the care they received and highlight areas requiring improvement. This systematic review is the first to comprehensively examine patient satisfaction with IPC interventions in hospital settings, providing valuable insights for clinicians, healthcare management and policymakers, particularly when 31% of IPC patients were not satisfied with their hospital stay. Greater involvement of patients and the public is increasingly recognised as essential in the prevention and control of HAIs.^65^ Enhancing patient engagement will help inform and improve IPC programmes. Studies on patient satisfaction can provide important insights into the patient experience and whether IPC interventions align with their expectations. Our findings suggest that greater PPI could improve patient experience with IPC. Potential approaches include co-developing IPC-specific satisfaction instruments with patients and the public; involving patients in the design of IPC-related educational materials and communication strategies related to IPC measures and including patient representatives in IPC committees or quality improvement initiatives. Such interventions could ensure that patient perspectives are embedded into IPC practices, and those institutional priorities are more closely aligned with patient concerns.

## Conclusion

This review shows that measuring patient satisfaction with IPC intervention is possible and increasingly relevant. Despite the associated complexities of IPC, often involving multicomponent approaches and diverse HAI-specific pathways, several studies attempted to assess patient experience in this context. Our findings show that suitable data collection methods are available. The meta-analysis showed that 31% of patients receiving IPC interventions were dissatisfied with their hospital experience, indicating room for improvement in service provision. These findings highlight the need for greater engagement with PPI groups and the development of patient-centred research and guidelines to inform future IPC practice. Establishing consensus on appropriate measurement tools and priority areas is essential to capture meaningful data and improve the quality and acceptability of IPC interventions.

## Supplementary material

10.1136/bmjopen-2025-103431 online supplemental file 1

10.1136/bmjopen-2025-103431 online supplemental file 2

10.1136/bmjopen-2025-103431 online supplemental file 3

## Data Availability

No data are available.
