# Supplementary material for: Patient satisfaction with infection prevention and control interventions in acute hospitals: a systematic review and meta-analysis
Source: BMJ Open. 2025 Dec 14;15(12):e103431. doi: 10.1136/bmjopen-2025-103431 (PMC12706194; doi:10.1136/bmjopen-2025-103431)
Supplement: online supplemental file 1 [file bmjopen-15-12-s001.docx]

**Supplementary material 1.** Search terms and search strings for each electronic source

| **Database** | **Infection control terms searched** | **Patient satisfaction terms searched** | **Limiters applied** |
| --- | --- | --- | --- |
| Medline | (MH "Infection Control") OR (MH "Disease Transmission, Infectious") OR "infection prevention*" OR "infection control*" OR "IPC" OR "Isolation precaution*" OR (MH "Cross Infection/PC") OR "hygiene" OR "hand hygiene" OR "environmental hygiene" OR "disinfection" OR "sterilisation" OR "sterilization" OR "personal protective equipment" OR "PPE" OR "standard precaution*" OR “contact precaution*” OR "transmission-based precautions" OR "transmission based precautions" OR “superbug*” | (MH "Patient Satisfaction") OR "Patient* Satisfaction*" OR "patient* experience*" OR "satisfaction survey*" OR "quality of care" OR "healthcare satisfaction" |  |
| Cinahl | (MH "Infection Control+") OR (MH "Infection Preventionists") OR (MH "Infection Control Nursing") OR (MH "Disease Transmission+") OR (MH "Hygiene") OR (MH "Handwashing+") OR (MH "Sterilization and Disinfection") OR (MH "Disinfectants") OR (MH "Personal Protective Equipment") OR (MH "Protective Clothing+") OR (MH "Ear Protective Devices") OR (MH "Protective Devices") OR (MH "Head Protective Devices") OR (MH "Eye Protective Devices") OR (MH "Respiratory Protective Devices") OR (MH "Universal Precautions") OR "infection control" OR “infection prevention*" OR "disease transmission" OR "hygiene" OR "environmental hygiene" OR "disinfection or sterilization or cleaning" OR "protective equipment" OR "standard precaution*" OR “contact precaution*” OR "transmission based precaution*" OR "transmission-based precaution*" OR “superbug*” | (MH "Patient Satisfaction+") OR (MH "Patients+") OR (MH "Quality of Health Care") OR "patient* satisfaction*" OR "patient* experience*" OR "healthcare satisfaction" OR "quality of care" | Peer Reviewed; Research Article; Exclude Pre-CINAHL; Exclude MEDLINE records |
| APA Psycinfo | "infection control" OR "infection prevention" OR "infection control and prevention" OR "ipc" OR “hygiene” OR "hand hygiene" OR DE "Disinfectants" OR "disinfection" OR "sterilization" OR “sterilisation” OR "cleaning" OR "isolation precaution*" OR "contact precaution*" OR "isolation precaution*" OR "personal protective equipment" OR "ppe" OR DE "Personal Protective Equipment" OR DE "Face Masks" OR "standard precaution*" OR DE "Disease Transmission" OR "transmission based precaution*" OR "transmission-based precaution*" OR DE "Hygiene" OR “superbug*” | "Patient Satisfaction"[Subject Heading] OR "patient* satisfaction*" OR "patient* experience*" OR "satisfaction survey*" OR "quality of care" OR "healthcare satisfaction" OR DE "Client Satisfaction" OR DE "Patient Centered Care" OR DE "Quality of Care" OR "quality of care" OR "healthcare satisfaction" |  |
| Scopus | TITLE-ABS-KEY (“infection control*" OR "disease transmission" OR "IPC" OR "infection prevention*" OR "hygiene" OR "hand hygiene" OR "environmental hygiene" OR "disinfection" OR "sterilisation" OR "sterilization" OR "isolation precaution*" OR "cross infection" OR "protective clothing" OR "personal protective equipment" OR "PPE" OR "standard precaution*" OR "contact precaution*" OR "transmission-based precautions" OR "transmission based precautions ” OR “superbug*”) | TITLE-ABS-KEY (“patient* satisfaction*" OR "patient* experience*" OR "satisfaction survey*" OR "quality of care" OR "healthcare satisfaction”) |  |
| Web of science | TS= ("infection control*" OR "disease transmission" OR "IPC" OR "infection prevention*" OR "hygiene" OR "hand hygiene" OR "environmental hygiene" OR "disinfection" OR "sterilisation" OR "sterilization" OR "isolation precaution*" OR "cross infection" OR "protective clothing" OR "personal protective equipment" OR "PPE" OR "standard precaution*" OR "contact precaution*" OR "transmission-based precautions" OR "transmission based precautions" OR “superbug*”) | TS= ("patient* satisfaction*" OR "patient* experience*" OR "satisfaction* survey*" OR "quality of care" OR "healthcare satisfaction") |  |
| Cochrane Library | #1 MeSH descriptor: [Infection Control] explode all trees  #2 MeSH descriptor: [Disease Transmission, Infectious] explode all trees  #3 MeSH descriptor: [Cross Infection] explode all trees  #4 MeSH descriptor: [Hygiene] explode all trees  #5 (infection NEXT/1 prevention*) OR (Infection NEXT/1 control*) OR “IPC” OR (Isolation NEXT/1 precaution*) OR (hygiene) OR (hand NEXT/1 hygiene) OR (environmental NEXT/1 hygiene) OR (disinfection) OR (sterilisation) OR (sterilization) OR (personal NEXT/1 protective NEXT/1 equipment) OR (PPE) OR (standard NEXT/1 precaution*) OR (contact NEXT/1 precaution*) OR (transmission-based NEXT/1 precautions) OR (transmission NEXT/1 based NEXT/1 precautions) OR (superbug*)  #1 OR #2 OR #3 OR #4 OR #5 | #7 MeSH descriptor: [Patient Satisfaction] explode all trees  #8 (Patient* NEXT/1 Satisfaction*) OR (patient* NEXT/1 experience*) OR (satisfaction NEXT/1 survey*) OR (quality NEXT/1 of NEXT/1 care) OR (healthcare NEXT/1 satisfaction)  #7 OR #8 |  |
| Embase | 'infection control'/de OR 'infection control*':ab,ti OR 'disease transmission'/de OR 'disease transmission':ab,ti OR 'ipc':ab,ti OR 'infection prevention*:ab,ti' OR 'hygiene'/de OR 'hygiene':ab,ti OR 'hand hygiene'/de OR 'hand hygiene':ab,ti OR 'environmental hygiene'/de OR 'environmental hygiene':ab,ti OR 'disinfection'/de OR 'disinfection':ab,ti OR 'sterilisation'/de OR 'sterilisation':ab,ti OR 'sterilization'/de OR 'sterilization':ab,ti OR 'isolation precaution*':ab,ti OR 'cross infection'/de OR 'cross infection':ab,ti OR 'protective clothing'/de OR 'protective clothing':ab,ti OR 'personal protective equipment'/de OR 'personal protective equipment':ab,ti OR 'ppe':ab,ti OR 'standard precaution*':ab,ti OR 'contact precaution*':ab,ti OR 'transmission-based precautions':ab,ti OR 'transmission based precautions':ab,ti OR 'superbug*':ab,ti | patient satisfaction'/de OR 'patient* satisfaction*’: ab, ti OR 'patient* experience*’: ab, ti OR 'satisfaction survey*':ab,ti OR 'quality of care':ab,ti OR 'healthcare satisfaction':ab,ti | NOT [medline]/lim |
