# Supplementary material for: Patient satisfaction with infection prevention and control interventions in acute hospitals: a systematic review and meta-analysis
Source: BMJ Open. 2025 Dec 14;15(12):e103431. doi: 10.1136/bmjopen-2025-103431 (PMC12706194; doi:10.1136/bmjopen-2025-103431)
Supplement: Supplementary file 4 [file bmjopen-2025-103431.reviewer_comments.pdf]

PEER REVIEW HISTORY

BMJ Open publishes all reviews undertaken for accepted manuscripts. Reviewers are asked to complete a checklist review form and are provided with free text boxes to elaborate on their assessment. These free text comments are reproduced below.

ARTICLE DETAILS

Title (Provisional)

Patient satisfaction with infection prevention and control interventions in acute hospitals: a systematic review and meta-analysis

Authors

Skally, Mairead; Kearney, Aoife; Strawbridge, Judith; Heritage, John; Cox, Cheryl; Bennett, Kathleen E; Humpreys, Hilary; Fitzpatrick, Fidelma

---

VERSION 1 - REVIEW

**Reviewer**	**1**
**Name**	**Ardura-Garcia, Cristina**
**Affiliation**	**University of Bern**
**Date**	**04-Jun-2025**
**COI**	**None**

This systematic review by Skally et al. aims to describe published studies on infection and prevention control (IPC) interventions, where patient satisfaction was reported. For this, they included 29 observational and interventional studies published between 2000-2024. They reported a patient satisfaction between 58-97% with IPC interventions, and a pooled satisfaction of 69% (95%CI 64-74%) for isolation precautions, with no comparison or control group.

The aim and scope of this study is relevant, as patient satisfaction is vital for IPC interventions as it may improve their implementation and uptake. However, I believe the methods used in this systematic review and meta-analysis do not enable an accurate assessment of patient satisfaction with IPC interventions, as I will develop in the comments.

General

1. The systematic search was done 1 year ago (June 2024). I believe this search should be now updated to include papers published in the last year.

2. Most of the studies included are patient satisfaction surveys performed on patients on whom an IPC measure was implemented (mostly isolation), but the aim of the study was not to evaluate patient satisfaction because of this intervention. They just report general patient satisfaction of patients who were isolated with no comparison with patients with similar conditions or who had undergone similar procedures but were not isolated. In this sense, the patient satisfaction reported is not a reflection of the IPC measure itself, but of the satisfaction

in general which will be a reflection of the particular hospital or ward, but not of the IPC measure.

3. Also, patient satisfaction in relation to isolation may depend a lot on the personal circumstances. In places where isolation means changing from a 4-5-bed shared room with only 1 bathroom to a private room with private bathroom, then patients may be very satisfied with this measure. On the other hand, patients who are completely isolated with no permitted or highly limited visits, may be very dissatisfied due to the loneliness associated to these situations. It is therefore difficult to interpret patient satisfaction with isolation as an IPC measure.

Introduction

4. The last paragraph of the introduction is not very clear to me, especially the sentence on lines 5-7. What are the authors trying to say here?

5. Aim: As I understand, what is relevant is the patient satisfaction WITH an IPC intervention, and not studies reporting on patient satisfaction AND IPC interventions, as two separate entities.

Methods

6. I would suggest including a short summary of the inclusion criteria in the text.

7. The authors have excluded studies including patients < 18 years ('old'). Could they please explain why they did so? I guess these studies would include patient satisfaction surveys completed by the patients carers, which is also very relevant and an important part of hospitals.

8. Please describe if the screening of titles/abstracts and full-texts were performed in duplicate or not. For the data extraction and risk of bias assessment process, please describe what was done with the 20% of full-texts for which data was extracted in duplicate: what happened if they found discrepancies? What was the a priori percentage of discrepancies that they set as acceptable? What happened if this percentage was higher than stipulated?

9. I would include the lines 45-54 in the paragraph under 'Data extraction', where the data extracted is mentioned.

10. As mentioned above, in order to assess the satisfaction with IPC measures, the pooled analysis should be an analysis of absolute or relative difference between the IPC and non-IPC cohorts, for each study, and not of the percentage of satisfaction for the IPC patients alone. This last measure is not informative for the aim of this review.

11. In addition to this, direct comparison of patient satisfaction proportion between isolated and non-isolated patients can lead to bias if not adjusted for confounders. Isolated patients may have a more severe or complicated disease which will inevitably impact their overall satisfaction (longer hospital stays, more intense medical care, etc.)

Results

12. I find this section too long for the reader. There is no need to describe one by one the included studies, as it becomes a boring list of details that can be found in the tables. I understand it is difficult to summarise studies that cannot be put together due to differences in study design, etc, but it is anyways necessary to summarise much more the findings for each of the IPC interventions, to include only one paragraph per intervention. I would definitely not describe the results of each of the studies included in the random-effects meta-analysis.

13. On page 25, line 14-18, the authors describe the study by Lupion-Mendoza et al, reporting an overall satisfaction, but then say that overall satisfaction was not reported? This is confusing.

14. Page 25 paragraph on lines 23-32, please describe the scale used (maximum score?) so the reader may understand the values reported, and what means a higher patient satisfaction (is 6.2 better than 4.8?).

15. Something to bear in mind when comparing scores between IPC and non-IPC patient satisfaction, is not only if there is a difference, but whether this difference is clinically relevant. For example, page 25, lines 16-50.

16. Studies that measured patient satisfaction comparing those who developed a surgical site infection vs those who did not, have nothing to do with IPC measures' patient satisfaction. Healthcare associated infections and meant to be prevented by IPC measures, but are not an IPC measure in itself. I would exclude these studies (page 27, last paragraph).

17. Page 29, lines 22-33: I would only include this study if we assume these patients were isolated, and if they were, I would include this study in the section 'Isolation precautions'.

18. Page 29, lines 38-49: I don't believe this study on smoking cessation has any association with IPC interventions.

Discussion

19. It is recommended to start the Discussion section with a summary of the results. Now, the first paragraph reads more as part of the introduction.

20. The main findings that the authors report (now second paragraph of the discussion section) are a bit misleading. First, they state that they identified 29 studies reporting patient satisfaction with an IPC intervention. This is not the case for most of the included studies, which refer to overall satisfaction among patients who had received an IPC intervention (but were not questioned about this intervention in particular). Second, they refer to only one results (pooled percentage of patient satisfaction for isolation measures) that reflects the findings of only 4 out of the 29 included studies, and they report that 69% is a relatively low level of satisfaction. Why is this a low level of satisfaction? What level of satisfaction would they expect to be good or high?

21. Limitations: I suggest being more specific about the quality of the included studies: What proportion were of low/moderate/high quality? How can this affect the results of the review?

22. I believe it will be much more useful to perform a review of qualitative studies on patient satisfaction concerning IPC measures in particular, as this will reflect in much more detail the patient's views.

**Reviewer**	**2**
**Name**	**Markwart, Robby**
**Affiliation**	**Jena University Hospital, Institute of General Practice and Family Medicine**
**Date**	**17-Jun-2025**
**COI**	**None**

The study titled 'Patient satisfaction with infection prevention and control interventions in acute hospitals: a systematic review and meta-analysis' by Skally et al. investigated patient experiences with IPC interventions and analysed the methods were used to measure patient satisfaction.

The problem I see with this study is that a significant body of evidence studied patient satisfaction not in the context of IPC but in the context of overall healthcare. This might introduce bias into the results, which is also briefly mentioned by the authors.

Overall, the study is well conducted and the results are clearly presented. I have some comments that may help to improve the manuscript.

Comments:

• Abstract: 'Thirty-one percent of isolated patients were not satisfied with their care' I think this conclusion is not supported by the evidence analysed in this study. This study investigated patient experiences with IPC, rather than their overall experience of healthcare. It is possible that patients are satisfied with their healthcare, but not with any IPC interventions they have experienced.

• Methods: This study does not include any qualitative studies on patient experiences of IPC interventions. Was the search strategy specifically designed to identify quantitative research? In my opinion, qualitative research would facilitate the acquisition of valuable insights into patient experiences of IPC measures.

• Methods / search strategy: How was the search strategy validated? I would have expected the use of more search terms (synonyms) to increase the sensitivity of the search. For example 'patient* attitude*', 'patient* opinion*', 'patient belief*', etc. One could also consider synonyms for 'patient', such as 'inpatient*', 'outpatient*', etc.

• Methods: What methods were used in the meta-analyses to calculate the summary measures and weights (e.g. DerSimonian and Laird (DL) method, etc.)?

• Discussion: ‘Additionally, the review highlights the need for better-tailored methods to measure patient satisfaction with IPC interventions, emphasizing the importance of patient and public involvement in developing these.’ Can the authors explain how their study ‘highlights the need for better-tailored methods to measure patient satisfaction with IPC interventions’? What are the ‘better-tailored methods’? As expected, this study shows that there is a large heterogeneity in IPC interventions studied and patient satisfaction assessment methods used. Do the authors advocate for more standardized methods?

• Discussion: ‘This showed a relatively low level of satisfaction at 69%.’ On the other side, more than two-third of the patients were satisfied with their care, which is actually quite high, when you consider that many patients underwent restrictive interventions, such as isolation.

• Discussion: The authors should discuss the methodological limitations of their study (e.g. sensitivity of the search, language bias, data extraction and risk of bias assessment not completely performed by two independent authors, etc.).

• Discussion: Given their knowledge and results, can the authors suggest any PPI interventions that could improve patient experience with IPC?

**Reviewer**	**3**
**Name**	**Olivier, Jake**
**Affiliation**	**University of New South Wales, School of Mathematics and Statistics**
**Date**	**24-Jul-2025**
**COI**	**None**

Interesting systematic review and meta-analysis on interventions for infection control and prevention. However, it is not clear in how the meta-analysis was conducted and how it has been reported.

The main outcome is the percentage of patients "satisfied" with infection prevention and control interventions. Among the included studies, they span several different study designs and several different methods for measuring satisfaction. Many studies appear to use Likert scales to measure satisfaction, e.g., strongly agree, agree, neutral, disagree, strongly disagree. How was this converted to "satisfaction" as a proportion?

The use of proportion satisfied is a reasonable outcome measure for the meta-analysis. However, the methods used to compute study-level confidence intervals should match that used for the overall proportion. Clopper-Pearson, which is known to be overly conservative, is used for the study-level estimates, and it is unclear what is used for the overall summary. The authors only state that they have been "pooled" using a random effects meta-analysis. Is it a weighted average using the reciprocal of study level variances plus the variance of the

random effect? That approach could be considered a "typical" meta-analysis approach, but Clopper-Pearson does not provide an estimate of variance for its estimate. So, the study-level information contained in the meta-analysis is not the same information that is reported at the study-level.

Next, it is not clear how the random effect was estimated in the meta-analysis. Was it maximum likelihood, restricted maximum likelihood, DerSimonian and Laird, or some other method?

How was heterogeneity tested? The I^2 statistic does not correspond to a hypothesis test, and so that is not where the p-value comes from. Is the p-value reported from Cochran's Q statistic? If so, this should be mentioned in the methods (along with more info about the meta-analysis in general).

Please order the studies in the forest plot in some reasonable way, e.g., by publication year.

Table 2. Listing out the countries or categorising them by continent would be much more informative than having "other" categories.

The results section seems to ramble on. Would a summary table or tables make more sense here?

Sentence spanning pages 33-34, different approaches will result in different results. So what? Perhaps explain why these results may be unrelated to the outcome being studied.

Conclusion. I cannot say I agree that service improvement is "badly" needed. Sure, there is room for improvement with 69% satisfaction, but I would not categorise that level as badly needing improvement.

VERSION 1 - AUTHOR RESPONSE

Reviewer 1: Dr. Cristina Ardura-Garcia, University of Bern

Overall comments

This systematic review by Skally et al. aims to describe published studies on infection and prevention control (IPC) interventions, where patient satisfaction was reported. For this, they included 29 observational and interventional studies published between 2000-2024. They reported a patient satisfaction between 58-97% with IPC interventions, and a pooled satisfaction of 69% (95%CI 64-74%) for isolation precautions, with no comparison or control group. The aim and scope of this study is relevant, as patient satisfaction is vital for IPC interventions as it may improve their implementation and uptake. However, I believe the methods used in this systematic review and meta-analysis do not enable an accurate assessment of patient satisfaction with IPC interventions, as I will develop in the comments.

RESPONSE: We thank Reviewer 1 for their constructive comments and the helpful feedback provided on our manuscript. We share their view that patient satisfaction is a vital component in the successful implementation of IPC interventions. We hope that our detailed responses to each of

their comments demonstrates that the methods used across the literature search, title screening, data collection, extraction and analysis, were appropriate and robust.

General comments:

1. The systematic search was done 1 year ago (June 2024). I believe this search should be now updated to include papers published in the last year

RESPONSE: We thank Reviewer 1 for this feedback on our manuscript. We acknowledge that some time has passed since the initial literature search, which was conducted prior to the manuscript's submission in early April 2025. At that time, the search captured the most current evidence available. The systematic review involved an extensive search across seven major databases and screening of over 13,000 titles, ensuring a thorough and representative synthesis. Given the scope and depth of the original search, we believe the findings remain timely and relevant. While we appreciate the suggestion to update the search, we respectfully consider that re-running it is unlikely to yield new evidence that would meaningfully alter the conclusions. We trust the Editor and Reviewer will take into account the methodological rigor and continued relevance of the review in its current form.

2. Most of the studies included are patient satisfaction surveys performed on patients on whom an IPC measure was implemented (mostly isolation), but the aim of the study was not to evaluate patient satisfaction because of this intervention. They just report general patient satisfaction of patients who were isolated with no comparison with patients with similar conditions or who had undergone similar procedures but were not isolated. In this sense, the patient satisfaction reported is not a reflection of the IPC measure itself, but of the satisfaction in general, which will be a reflection of the particular hospital or ward, but not of the IPC measure.

RESPONSE: We thank the reviewer for this observation. We acknowledge the relevance of this point in the context of eight studies addressing general hygiene practices. However, we respectfully believe that it does not apply to the remaining 21 studies included in our review. To clarify this distinction, we have addressed the issue in the manuscript and supported our position with relevant guidelines on standard precautions issued (2) and core components of IPC Programmes (4) by the WHO. This clarification can be found in Methods, Selection Processes on pages 8 and 9, lines 177 to 179 where we state:

> *"Studies reporting on patient satisfaction and general hygiene were assessed for eligibility as the cleanliness of the hospital environment is recognised as a key component of IPC in healthcare settings.[2, 4]"*

In the revised version of our manuscript following Reviewer 1's observations, we have further clarified our objective of capturing patient satisfaction outcomes specifically in the context of IPC interventions or outcomes closely aligned with IPC practices. This clarification has been incorporated into the Abstract on page 3, line 52:

> ***"Objectives:*** *This systematic review aims to identify peer-reviewed publications reporting patient satisfaction outcomes in the context of IPC interventions, to document the methods used to quantify patient satisfaction and to conduct a meta-analysis on reported satisfaction levels"*

We have also incorporated this clarification into the Introduction on page 6, lines 118 to 123 as follows:

> "*This systematic review aimed to identify peer-reviewed publications reporting patient satisfaction outcomes in the context of implemented IPC interventions, whether satisfaction was the primary focus or one of several factors examined, to document the methods used to quantify patient satisfaction and to conduct a meta-analysis of reported satisfaction levels* associated with specific *IPC interventions where* feasible"

Likewise, we have also incorporated this clarification into the Selection process of the Methods section on page 9, lines 179 to 182:

> "*Studies were eligible if they reported patient satisfaction in the context of implemented IPC interventions, or in relation to IPC-related outcomes (e.g., surgical site infection (SSI)), given the direct association between these outcomes and IPC effectiveness."*

Lastly, we have incorporated this clarification into the Discussion, on page 25, lines 458 to 459:

> "*This systematic review identified 29 studies reporting patient satisfaction* outcomes in *the context of IPC interventions"*

3. Also, patient satisfaction in relation to isolation may depend a lot on the personal circumstances. In places where isolation means changing from a 4-5-bed shared room with only 1 bathroom to a private room with private bathroom, then patients may be very satisfied with this measure. On the other hand, patients who are completely isolated with no permitted or highly limited visits, may be very dissatisfied due to the loneliness associated to these situations. It is therefore difficult to interpret patient satisfaction with isolation as an IPC measure.

RESPONSE: We thank the reviewer for another helpful comment. We fully acknowledge that patients may experience isolation differently, and this was carefully considered during the study design phase, and in discussions with our PPI contributors. The issues raised by Reviewer 1 pertain to "patient experience" whereas the primary objective of this systematic review was to examine "patient satisfaction". We believe that focusing on patient satisfaction enables more consistent and standardised data capture across large patient populations. This approach empowers patients to assess whether their care met expectations and provides valuable insights for decision makers to identify areas for improvement. To clarify this distinction, we have added the following statement to the Introduction on page 5, lines 103 to 110:

> "*Assessing whether patient expectations are met is key to capturing the patient voice. Two commonly used measures are 'patient experience' and 'patient satisfaction'. Although often used interchangeably, they represent distinct concepts. Patient experience reflects patients' views of care relative to their expectations, while patient satisfaction measures whether or not those expectations were met. Qualitative studies of patient experience may provide deeper insights into perceptions of care, whereas, quantitative satisfaction measures provide ratings that allow for systematic synthesis where methods and measures are comparable"*

4. The last paragraph of the introduction is not very clear to me, especially the sentence on lines 5-7. What are the authors trying to say here?

RESPONSE: We thank Reviewer 1 for highlighting this point for clarification. This systematic review sought to identify studies reporting patient satisfaction outcomes in the context of implemented IPC measures, regardless of whether satisfaction with IPC was the primary focus or one of several factors examined (such as general hygiene practices). To clarify this, we have revised the text of the Introduction accordingly on page 6, lines 114 to 123 as follows:

> *"So, how satisfied are patients with IPC services? Previous systematic reviews have focused either on 'patient satisfaction' or on only one IPC measure. To date no systematic review has addressed patient satisfaction with IPC interventions, whether assessed directly or as part of broader factors such as general hygiene. As such, it is not known whether patients' expectations of IPC measures are being met. This systematic review aimed to identify peer-reviewed publications reporting patient satisfaction outcomes in the context of implemented IPC interventions, whether satisfaction was the primary focus or one of several factors examined, to document the methods used to quantify patient satisfaction and to conduct a meta-analysis of reported satisfaction levels associated with specific IPC interventions where feasible."*

5. Aim: As I understand, what is relevant is the patient satisfaction WITH an IPC intervention, and not studies reporting on patient satisfaction AND IPC interventions, as two separate entities.

RESPONSE: We thank the reviewer for this comment, which we consider aligns with point 2 addressed above. The inclusion criterion for this review was patient satisfaction in the context of IPC measures, rather than inclusion limited to studies focusing exclusively on satisfaction with IPC. This broader approach allowed us to capture general satisfaction outcomes while still highlighting the potential influence of IPC measures on patient perceptions. Our response to this issue is addressed under Point 4 above.

Methods

6. I would suggest including a short summary of the inclusion criteria in the text.

RESPONSE: We thank the reviewer for this helpful feedback. As this point was also raised by the Editor under Point 3, we have amended the text accordingly as described in our response to that point.

7. The authors have excluded studies including patients < 18 years ('old'). Could they please explain why they did so? I guess these studies would include patient satisfaction surveys completed by the patients' carers, which is also very relevant and an important part of hospitals.

RESPONSE: We thank Reviewer 1 for this important comment. We limited our search to studies involving adult patients (≥18 years), as no prior systematic review has addressed this topic. We considered it important to first establish a baseline by focusing on surveys in which patients could directly self-report their experiences, something less feasible in paediatric populations. As noted by Reviewer 1, satisfaction in paediatric settings is more commonly reported by carers. While we fully agree that carer perspectives are highly relevant, particularly in paediatric care, we chose to focus this review on adult populations as a first step. We believe future research could build on this work

by extending the approach to include children and carers, thereby providing a more comprehensive understanding. To address Reviewer 1's point we have added the following clarification to the Discussion on page 30, lines 577 to 580:

> *"Further research should be extended to include the perspectives of children and carers to provide a more comprehensive understanding and overview of patients experience across diverse populations and healthcare settings."*

8. Please describe if the screening of titles/abstracts and full-texts were performed in duplicate or not. For the data extraction and risk of bias assessment process, please describe what was done with the 20% of full-texts for which data was extracted in duplicate: what happened if they found discrepancies? What was the a priori percentage of discrepancies that they set as acceptable? What happened if this percentage was higher than stipulated?

RESPONSE: Thank you for raising this helpful point for clarification. Titles/abstracts and full text screening was performed in duplicate. For 20% of full texts, data extraction and assessment were also performed in duplicate, with discrepancies resolved through discussion and, where necessary, arbitration by a third reviewer. While no a priori discrepancy threshold was set; this process served as a quality assurance measure to ensure consistency before proceeding with single extraction. To clarify this, we have added the following text to the Methods of the manuscript on page 8, lines 176 to 177:

> *"…. assessed for eligibility, as outlined above, independently by two reviewers, MS and AK."*

We have also added the following text to page 9, lines 187 to 190:

> *"Discrepancies were resolved* through *discussion, with* arbitration by a *third reviewer (HH) if needed. No a priori discrepancy threshold was set as this process served as a quality check to ensure consistency* prior to single data extraction*."*

9. I would include the lines 45-54 in the paragraph under 'Data extraction', where the data extracted is mentioned.

RESPONSE: We thank Reviewer 1 for highlighting this point. We have renamed this section from 'Data synthesis' to 'Data Extraction' on page 9, line 185.

Additionally, we have specified the software used for this process on page 9, line 190:

> *"Data was extracted into a Microsoft Excel file (MS) and included title, authors, year, journal, study design, intervention, data capture…."*

10. As mentioned above, in order to assess the satisfaction with IPC measures, the pooled analysis should be an analysis of absolute or relative difference between the IPC and non-IPC cohorts, for each study, and not of the percentage of satisfaction for the IPC patients alone. This last measure is not informative for the aim of this review.

RESPONSE: We thank Reviewer 1 for this insightful comment. We agree that, ideally, patient satisfaction with IPC measures should be assessed by comparing absolute or relative differences between IPC and non-IPC cohorts. However, in most studies included in our review, comparator (non-IPC) groups were either absent or not reported in a sufficiently consistent manner to support meta-analysis of between-group differences. As a result, we were only able to pool satisfaction

proportions from IPC cohorts. Where comparator data were available, we have included these results in Supplementary material 2 for reference. This systematic review highlights the need for future studies to report comparator satisfaction data more consistently, to enable more robust synthesis of the relative impact of IPC measures on patient satisfaction. We have revised the manuscript to acknowledge more explicitly this limitation in both the Methods and Discussion sections as follows:

The revision to the Data synthesis of the Methods section is on page 10, lines 207 to 210:

> *"Due to inconsistent reporting of comparator data across studies, a quantitative synthesis of absolute or relative differences between IPC and non-IPC cohorts was not feasible. For studies where comparator data were available, the results have been presented within the main dataset.*

And to the Discussion, on page 29, lines 567 to 570, we have added:

> "*Most studies did not report satisfaction outcomes for non-IPC comparator groups in a manner that allowed for the calculation of absolute or relative differences. As a result, the analysis focused on pooled satisfaction proportions within IPC cohorts rather than comparative effect estimates."*

11. In addition to this, direct comparison of patient satisfaction proportion between isolated and non-isolated patients can lead to bias if not adjusted for confounders. Isolated patients may have a more severe or complicated disease which will inevitably impact their overall satisfaction (longer hospital stays, more intense medical care, etc.)

RESPONSE: We thank the reviewer for raising this important methodological consideration. We agree that direct comparisons between isolated and non-isolated patients may be subject to confounding. However, assessment of adjusted comparative effects was beyond the primary aim of our review. Our objective was to synthesise reported satisfaction outcomes in the context of IPC measures and to document the methods used to quantify these outcomes. As only a small number of included studies reported adjusted analyses, our synthesis reflects unadjusted crude satisfaction estimates. To clarify this limitation, we have added the following statement to the Discussion of the manuscript on page 27, lines 503 to 507:

> "*As most studies reported only crude satisfaction estimates of patient satisfaction, direct comparisons between isolated and non-isolated patient groups were not conducted. Any such comparative analysis would need adjustment for potential confounders such as illness severity, length of hospital stay and other relevant clinical factors."*

Results

12. I find this section too long for the reader. There is no need to describe one by one the included studies, as it becomes a boring list of details that can be found in the tables. I understand it is difficult to summarise studies that cannot be put together due to differences in study design, etc, but it is anyways necessary to summarise much more the findings for each of the IPC interventions, to include only one paragraph per intervention. I would definitely not describe the results of each of the studies included in the random-effects meta-analysis.

RESPONSE: We appreciate this constructive feedback. Similar feedback was also raised by Dr. Jake Olivier (reviewer 3, point 8 below). In response, we have extensively reduced the Results section, pages 11 to 24, focusing on the most relevant findings and removing supplementary detail. All changes have been tracked throughout the revised Results section and are included as an Appendix for reference.

13. On page 25, line 14-18, the authors describe the study by Lupion-Mendoza et al, reporting an overall satisfaction, but then say that overall satisfaction was not reported? This is confusing.
RESPONSE: Thanks for highlighting this to us. We have revised and clarified the relevant of the Results section on pages 18 to 19, lines 309 to 312:

> "*Lupión-Mendoza et al. (2015, high quality) reported 93.1% satisfaction with 'care received from professionals' in Spain, though overall satisfaction of the hospital and scale details were not provided*"

14. Page 25 paragraph on lines 23-32, please describe the scale used (maximum score?) so the reader may understand the values reported, and what means a higher patient satisfaction (is 6.2 better than 4.8?).

RESPONSE: Thank you for highlighting this oversight. We have revised the Results of the manuscript accordingly and the text has been amended on page 19, lines 314 to 323 as follows:"

> *"Gaube et al. (2023, high quality), evaluated patient dissatisfaction associated with isolation precautions using the Picker Patient Experience Questionnaire[28] This employs a dichotomous scoring method to generate a 'problem score', whereby responses are coded as either 0 or 1 and subsequently summed. The higher the score, the greater the patient dissatisfaction. Among isolated patients (n=118) the mean problem score was 6.20 (standard deviation (SD) 4.08), compared with a mean score of 4.85 (SD 3.11) reported by non-isolated patients".*

15. Something to bear in mind when comparing scores between IPC and non-IPC patient satisfaction, is not only if there is a difference, but whether this difference is clinically relevant. For example, page 25, lines 16-50.
RESPONSE: We thank reviewer 1 for this helpful observation. We agree that evaluating the clinical relevance of differences in patient satisfaction scores is an important consideration. However, as noted in our response to point 10, this type of assessment was beyond the scope of our review. Our primary aim was to synthesis reported patient satisfaction outcomes and methods used in the context of IPC measures. Most included studies did not define thresholds for what would constitute a clinically meaningful difference in satisfaction scores. Consequently, we limited our synthesis to reporting absolute values and proportions as presented in the original studies. We have added a clarification to this effect in the Discussion on page 27, lines 503 to 507:

> *"As most studies reported only crude satisfaction estimates of patient's satisfaction, direct comparisons between isolated and non-isolated patients were not conducted. Any such comparative analysis would adjustment for potential confounders such as illness severity, length of hospital stay and other relevant clinical factors."*

16. Studies that measured patient satisfaction comparing those who developed a surgical site infection vs those who did not, have nothing to do with IPC measures' patient satisfaction. Healthcare associated infections and meant to be prevented by IPC measures, but are not an IPC measure in itself. I would exclude these studies (page 27, last paragraph).

RESPONSE: We thank Reviewer 1 for this observation. Our inclusion criteria were designed to encompass studies reporting patient satisfaction outcomes in the context of IPC interventions or outcomes directly attributable to IPC practices. Although surgical site infection (SSI) is not itself an IPC intervention per se, it represents a key outcome that IPC measures specifically aim to prevent. We therefore considered patient satisfaction related to the prevention of surgical site infection to be relevant, as it offers indirect but relevant insight into patient perceptions of IPC effectiveness. To clarify this point, we have added the following statement to the Selection process of the Methods section on page 9, lines 179 to 182:

> *"Studies were eligible if they reported patient satisfaction in the context of implemented IPC interventions, or in relation to IPC-related outcomes (e.g., surgical site infection (SSI)), given the direct association between these outcomes and IPC effectiveness "*

We have also expanded the Discussion to address this point. The following text has been added on pages 28, lines 542 to 545:

> *"Several included studies assessed patient satisfaction in with the context of SSI prevention. While SSI represents an outcome rather than a direct IPC intervention, it is a core endpoint of IPC programs and hence provides indirect insight into patient perceptions of IPC effectiveness, though the findings should be interpreted within this context."*

17. Page 29, lines 22-33: I would only include this study if we assume these patients were isolated, and if they were, I would include this study in the section 'Isolation precautions.

RESPONSE: We thank Reviewer 1 for this perspective. This study titled *"Effect of contact precautions for MRSA on patient satisfaction scores"*, specifically evaluated whether the implementation of contact precautions for patients with a history of MRSA influenced HCAHPS satisfaction scores. As such, we considered this study to be directly aligned with our primary objective of capturing patient satisfaction outcomes in the context of IPC interventions or outcomes closely linked to IPC practices. For this reason, we retained the study in our analysis as originally reported, rather than relocating it to the subsection on isolation precautions or excluding it altogether.

18. Page 29, lines 38-49: I don't believe this study on smoking cessation has any association with IPC interventions.
RESPONSE: Thank you for this observation. This study titled *"Feasibility and patient satisfaction with smoking cessation interventions for prevention of healthcare-associated infections in inpatients"* evaluated patient engagement, satisfaction, and withdrawal symptoms in the context of a smoking cessation program explicitly implemented to reduce the risk of healthcare-associated infections (HAIs). As the intervention was framed and delivered as an IPC strategy, we considered the study to fall within our inclusion criteria and relevant to our review objective of capturing patient satisfaction outcomes related to IPC measures.

Discussion

19. It is recommended to start the Discussion section with a summary of the results. Now, the first paragraph reads more as part of the introduction.
RESPONSE: Thank you for this observation. In response, we have revised the Discussion section by relocating the relevant paragraph further into the text. The discussion now begins with a summary

of results, which was originally presented as the second paragraph of the discussion on page 25, lines 458 to 463:

> *"This systematic review identified 29 studies reporting patient satisfaction outcomes in the context of IPC interventions. The findings revealed considerable heterogeneity, both in the types of IPC interventions studied, and in the methodologies used to assess satisfaction, including survey instruments and rating scales used. Meta-analysis was possible for only four studies that used the HCAHPS survey to assess patients' views during isolation. These studies showed a satisfaction level of 69%, indicating a relatively low level of satisfaction among isolated patients".*

Our original first paragraph is now moved to pages 30 and 31, lines 587 to 600 has been revised as follows:

> *"This systematic review is the first to comprehensively examine patient satisfaction with IPC interventions in hospital settings, providing valuable insights for clinicians, healthcare management and policymakers, particularly when 31% of IPC patients were not satisfied with their hospital stay. Greater involvement of patients and the public is increasingly recognised as essential in the prevention and control of HAIs.[55] Enhancing patient engagement will help inform and improve IPC programmes. Studies on patient satisfaction can provide important insights into the patient experience and whether IPC interventions align with their expectations. Our findings suggest that greater PPI could improve patient experience with IPC. Potential approaches include co-developing IPC-specific satisfaction instruments with patients and the public; involving patients in the design of IPC-related educational materials and communication strategies related to IPC measures; and including patient representatives in IPC committees or quality improvement initiatives. Such interventions could ensure that patient perspectives are embedded into IPC practices and those institutional priorities are more closely aligned with patient concerns."*

20. The main findings that the authors report (now second paragraph of the discussion section) are a bit misleading. First, they state that they identified 29 studies reporting patient satisfaction with an IPC intervention. This is not the case for most of the included studies, which refer to overall satisfaction among patients who had received an IPC intervention (but were not questioned about this intervention in particular). Second, they refer to only one results (pooled percentage of patient satisfaction for isolation measures) that reflects the findings of only 4 out of the 29 included studies, and they report that 69% is a relatively low level of satisfaction. Why is this a low level of satisfaction? What level of satisfaction would they expect to be good or high?

RESPONSE: We thank Reviewer 1 for this observation. We acknowledge that the original phrasing of our main findings may have inadvertently suggested that all 29 included studies explicitly measured satisfaction with IPC interventions. In fact, the majority of studies reported overall patient satisfaction among populations exposed to IPC measures, rather than satisfaction with the measures themselves.

To address this, we have revised the Objectives in the Abstract on page 3 lines 51 to 54, to more accurately reflect the included studies:

“This systematic review aims to identify peer-reviewed publications reporting patient satisfaction outcomes in the context of IPC interventions, to document the methods used to assess patient satisfaction and to conduct a meta-analysis on reported satisfaction outcomes.”

We have also revised the Introduction on page 6 lines 118 to 121 as follows:

“This systematic review aimed to identify peer-reviewed publications reporting patient satisfaction outcomes in the context of implemented IPC interventions, whether satisfaction was the primary focus or one of several factors examined, to document…..”

Likewise, we have revised the Selection process in the Methods section on page 9 lines 179 to 182:

“Studies were eligible if they reported patient satisfaction in the context of implemented IPC interventions, or in relation to IPC-related outcomes (e.g., surgical site infection(SSI)), given the direct association between these outcomes and IPC effectiveness.”

Finally, we have revised the Discussion on page 25 lines 458 to 463:

“This systematic review identified 29 studies reporting patient satisfaction outcomes in the context of IPC interventions. The findings revealed considerable heterogeneity, both in the types of IPC interventions studied, and in the methodologies used to assess satisfaction, including survey instruments and rating scales used. Meta-analysis was possible for only four studies that used the HCAHPS survey to assess patients’ views during isolation. These studies showed a satisfaction level of 69%, among isolated patients.”

In addition, we have expanded the Discussion on the topic of acceptable rates of patient satisfaction. This addition is on page 27, lines 516 to 521:

“Patient satisfaction with hospital inpatient care is typically assessed using standardised national surveys, with satisfaction targets for positive overall ratings often set at or above 80%. For example, Ireland's 2024 national inpatient survey reported that 85% of respondents rated their experience as 'good' or 'very good,' consistent with international benchmarks. Likewise, top performing hospitals in the US consistently achieve patient satisfaction rates in the 80–85% range as measured by the HCAHPS survey.”

Regarding the pooled analysis, we acknowledge that our initial description of the 69% satisfaction rate as “relatively low” may have lacked sufficient justification. To address this, we have rephrased the statement to present the result in a more neutral manner. These revisions have been made:

Firstly, in our Conclusion in the Abstract on page 4, lines 79 to 80:

*“**Conclusion**: Sixty-nine percent of isolated patients reported satisfaction with their care*

In the Discussion on page 25, lines 463 to 464:

“*These studies reported a satisfaction level at 69% among isolated patients.”*

And lastly in the Conclusion on pages 31 , lines 608 to 614:

“The meta-analysis showed that 31% of patients receiving IPC interventions were dissatisfied with their hospital experience, indicating room for improvement in service provision. These findings highlight the need for greater engagement with PPI groups and

the development of patient-centred research and guidelines to inform future IPC practice. Establishing consensus on appropriate measurement tools and priority areas is essential to capture meaningful data and improve the quality and acceptability of IPC interventions.

21. Limitations: I suggest being more specific about the quality of the included studies: What proportion were of low/moderate/high quality? How can this affect the results of the review?

RESPONSE: We thank the reviewer for this useful suggestion. These results are presented in the Results section on page 17, lines 261 to 266 under "Risk of bias and overall quality of evidence "

In response to this comment, we have also expanded the Discussion section to reflect on this topic in greater depth, on page 27, lines 501 to 507:

"*The quality of the included studies was mostly high (86%). While the presence of moderate/low-quality studies introduces potential risk of bias in results and findings, this did not affect the findings of the meta-analysis, as these studies were excluded from the pooled analyses. As most studies reported only crude satisfaction estimates of patient satisfaction, direct comparisons between isolated and non-isolated patient groups were not conducted. Any such comparative analysis would need adjustment for potential confounders such as illness severity, length of hospital stay and other relevant clinical factors."*

And again, on pages *29, lines 570 to 571:*

"The overall quality of 14% of studies was rated as moderate however, these results were excluded from pooled analysis"

22. I believe it will be much more useful to perform a review of qualitative studies on patient satisfaction concerning IPC measures in particular, as this will reflect in much more detail the patient's views.

RESPONSE: Thank you for this view. We agree that a review of qualitative studies would offer valuable and detailed insights into patients' perspectives on IPC measures. However, the aim of the present review was to synthesise quantitative outcomes of patient satisfaction in the context of IPC interventions, to document the methods used to measure satisfaction, and, where feasible, to conduct meta-analyses of satisfaction levels. This focus was chosen to address a gap in the existing literature, as highlighted in the Introduction, given that previous reviews have either examined patient satisfaction in general or focussed on individual IPC measures. To our knowledge, none have synthesised quantitative satisfaction outcomes across a range of IPC interventions. To clarify this, we have added the following text to the Introduction on page 5, lines 107 to 110:

"Qualitative studies of patient experience may provide deeper insights into perceptions of care, whereas quantitative satisfaction measures provide ratings that allow for systematic synthesis where methods and measures are comparable."

Likewise, we have added the following text to the Discussion, on page 30, lines 583 to 586:

"*While questionnaire surveys lack detail compared with qualitatively assessing patient experience, they provide a consistent method of data capture from large numbers of patients and allow for systematic synthesis of findings where methods and measures are comparable"*

Reviewer 2: Dr. Robby Markwart, Jena University Hospital

Comments to the Author:

The study titled 'Patient satisfaction with infection prevention and control interventions in acute hospitals: a systematic review and meta-analysis' by Skally et al. investigated patient experiences with IPC interventions and analysed the methods were used to measure patient satisfaction.

The problem I see with this study is that a significant body of evidence studied patient satisfaction not in the context of IPC but in the context of overall healthcare. This might introduce bias into the results, which is also briefly mentioned by the authors.

Overall, the study is well conducted, and the results are clearly presented. I have some comments that may help to improve the manuscript.

RESPONSE:

We thank Reviewer 2 for the constructive feedback. The concern raised regarding the scope of the evidence, specifically that patient satisfaction was assessed in the context of overall healthcare rather than specifically in relation to IPC is noted. We believe that this issue, along with other related concerns, have been addressed through the corresponding revisions to the manuscript in response to points 2, 4, 15, 16, 19, 20 and 22 raised by Reviewer 1 . The amended text under these points incorporates the reviewers' suggestions and aims to enhance the clarity of our manuscript.

Specific Comments:

Abstract:

1. Thirty-one percent of isolated patients were not satisfied with their care' I think this conclusion is not supported by the evidence analysed in this study. This study investigated patient experiences with IPC, rather than their overall experience of healthcare. It is possible that patients are satisfied with their healthcare, but not with any IPC interventions they have experienced.

RESPONSE: We thank Reviewer 2 for this comment. These concerns have been addressed through the revisions made in response to Reviewer 1, under points 2, 10, 11, 19, 20 and 22. We believe that the responses and corresponding amendments under these points address the issue above highlighted by Reviewer 2.

Methods:

2. This study does not include any qualitative studies on patient experiences of IPC interventions. Was the search strategy specifically designed to identify quantitative research? In my opinion, qualitative research would facilitate the acquisition of valuable insights into patient experiences of IPC measures.

RESPONSE: We thank Reviewer 2 for this comment. The issue raised has been addressed in our response to Reviewer 1, specifically under point 22 We believe the revisions made in relation to this point adequately respond to the above concern and improve our manuscript.

3. Methods / search strategy: How was the search strategy validated? I would have expected the use of more search terms (synonyms) to increase the sensitivity of the search. For example

'patient* attitude*', 'patient* opinion*', 'patient belief*', etc. One could also consider synonyms for 'patient', such as 'inpatient*', 'outpatient*', etc.

RESPONSE: We thank Reviewer 2 for this helpful observation regarding our search strategy. In relation to the first point, the search strings for each database were developed in conjunction with an information specialist librarian. We subsequently piloted the initial search to ensure that it successfully retrieved key sentinel articles known to the research team, and cross-checked results across multiple databases to confirm consistency.

Regarding the use of suggested synonyms - our review was deliberately focused on the construct of patient satisfaction, rather than broader constructs such as attitudes, beliefs, or experiences. While expanding the search terms could have increased sensitivity, it would have reduced specificity and retrieved studies outside the scope of our review. Our published protocol is cited in the manuscript (Reference 21) to provide readers with further detail regarding the search strategy, should they wish to consult it. Given our objective to document quantitative assessments of patient satisfaction, we considered this term the most precise and standardised for capturing data aligned with our aims.

4. What methods were used in the meta-analyses to calculate the summary measures and weights (e.g., DerSimonian and Laird (DL) method, etc.)?

RESPONSE: We used Stata's metaprop routine to perform the meta-analysis, which applies the Wilson score method for calculating confidence intervals. To clarify this, we have added a statement to the Statistical Analysis section of the Methods, along with a supporting reference (24), on page 10, lines 214 to 217:

> *"Proportions were pooled using Stata's metaprop routine, applying the Freeman–Tukey double-arcsine transformation and a DerSimonian–Laird random-effects model; estimates were back-transformed to the proportion scale. Study-level 95% confidence intervals (CIs) used the Wilson (score) method.[24]"*

Reference Nyaga VN, Arbyn M, Aerts M. Metaprop: a Stata command to perform meta-analysis of binomial data. Archives of Public Health. 2014;72(1):39.

Discussion:

5. 'Additionally, the review highlights the need for better-tailored methods to measure patient satisfaction with IPC interventions, emphasizing the importance of patient and public involvement in developing these.' Can the authors explain how their study 'highlights the need for better-tailored methods to measure patient satisfaction with IPC interventions'? What are the 'better-tailored methods'? As expected, this study shows that there is a large heterogeneity in IPC interventions studied and patient satisfaction assessment methods used. Do the authors advocate for more standardized methods?

RESPONSE: We thank Reviewer 2 for raising this important point for clarification. Our review identified considerable heterogeneity both in the IPC interventions studied and, in the instruments, used to measure patient satisfaction. Many studies employed general satisfaction surveys that were not specifically designed to capture patients' views on IPC-specific elements (e.g., hand hygiene, isolation precautions, communication about infection risks). This limits both the comparability of findings across studies and the ability to draw conclusions about patient perspectives on IPC

interventions specifically. By "better-tailored methods" we refer to the need for IPC-specific patient satisfaction measures, ideally co-designed with patients, to improve relevance. We also advocate for greater standardisation of measurement tools to enhance comparability across studies. To clarify this issue, we have added the following text to the Discussion on pages 26 to 27, lines 483 to 499:

> *"Our findings highlight that many studies relied on generic patient satisfaction tools that may not adequately capture perceptions of IPC-specific practices. There is a need to develop tailored tools that include domains such as hand hygiene, isolation procedures, and communication regarding infection risk. Ideally these tools should be co-designed with patients and members of the public to ensure greater relevance and responsiveness to their concerns. Greater standardisation of IPC-specific satisfaction measures would also facilitate comparability across studies and healthcare settings Additionally, the review highlights the importance of PPI in the development of tailored tools to measure patient satisfaction with IPC interventions. Future surveys should be designed with meaningful input from patients to ensure that the data captured reflects their experiences and priorities, thereby enabling actionable conclusions. Future studies should focus on issues that concern patients, and not only on those perceived important by IPC teams. Some studies may need to address general IPC concerns, e.g., hospital hygiene, hand hygiene and PPE use. However, others should target specific issues, infections or settings, such as SSI, CDI and care in oncology/haematology units.*

6. 'This showed a relatively low level of satisfaction at 69%.' On the other side, more than two-third of the patients were satisfied with their care, which is actually quite high, when you consider that many patients underwent restrictive interventions, such as isolation.

RESPONSE: We thank Reviewer 2 for this comment. This point has been addressed in our response to Reviewer 1,specifically under point 20. We believe the revisions made in relation to this point have addressed the feedback raised.

7. The authors should discuss the methodological limitations of their study (e.g. sensitivity of the search, language bias, data extraction and risk of bias assessment not completely performed by two independent authors, etc.).

RESPONSE: We thank Reviewer 2 for this feedback. As noted in point 3 above, our search strategy was designed in collaboration with a specialist librarian to include broad terms, and we did not apply any language restrictions. This approach was intended to minimise the risk of missed relevant studies due to sensitivity or language bias. However, we acknowledge the importance of recognising other methodological limitations. In the revised manuscript, we now note that data extraction and risk of bias assessment were not conducted independently by two reviewers for all studies, which may introduce some potential for error or bias. This clarification has been added to the Discussion section on page 29, lines 560 to 571:

> *"Strengths of this study include the use of a rigorous methodology, adherence to the JBI and PRISMA guidelines, and a thorough critical appraisal of included studies. To minimise the risk of missing relevant publications broad search terms were applied across multiple databases without language restrictions. However, as data extraction and risk of bias assessment were not conducted in full duplicate for all studies, a small potential for error or bias should be considered when interpreting the findings. Additionally, the scope of meta-analysis was*

limited by the heterogeneity in both IPC interventions reported and patient satisfaction measurement methods. Most studies did not report satisfaction outcomes for non-IPC comparator groups in a manner that allowed for the calculation of absolute or relative differences. As a result, the analysis focused on pooled satisfaction proportions within IPC cohorts rather than comparative effect estimates. The overall quality of 14% of studies was rated as moderate however these results were excluded from pooled analysis."

8. Given their knowledge and results, can the authors suggest any PPI interventions that could improve patient experience with IPC?

RESPONSE: We thank Reviewer 2 for this helpful suggestion. While our review did not identify specific PPI interventions aimed at improving patient experience with IPC, we agree that our finding highlight the value of actively involving patients in the design and implementation of IPC strategies. To support this point and propose potential approaches, we have added the following text to the Discussion on pages 30 to 31, lines 594 to 600:

> *"Our findings suggest that greater PPI could improve patient experience with IPC. Potential approaches include co-developing IPC-specific satisfaction instruments with patients and the public; involving patients in the design of IPC-related educational materials and communication strategies related to IPC measures; and including patient representatives in IPC committees or quality improvement initiatives. Such interventions could ensure that patient perspectives are embedded into IPC practices and those institutional priorities are more closely aligned with patient concerns"*

Reviewer 3: Dr. Jake Olivier, University of New South Wales

Comments to the Author:

1. Interesting systematic review and meta-analysis on interventions for infection control and prevention. However, it is not clear in how the meta-analysis was conducted and how it has been reported.
RESPONSE: We thank Reviewer 3 for this positive overview. The meta-analysis processes have been clarified above in response to Reviewer 2, point 4 and further elaborated below under point 5 (in response to Reviewer 3).

Specific Comments:

2. The main outcome is the percentage of patients "satisfied" with infection prevention and control interventions. Among the included studies, they span several different study designs and several different methods for measuring satisfaction. Many studies appear to use Likert scales to measure satisfaction, e.g., strongly agree, agree, neutral, disagree, strongly disagree. How was this converted to "satisfaction" as a proportion?

RESPONSE We thank Reviewer 3 for raising this important point. In our review, we did not apply a uniform cut-off to define "satisfaction." Instead, we extracted and reported proportions as defined

by the original study authors, based on their chosen instruments and criteria (e.g., dichotomised Likert scales or predefined thresholds for satisfaction).

To clarify this point, we have added the following text to the Data synthesis of the Methods section on pages 9 to 10, lines 203 to 205:

> *"For each study, the number of satisfied patients were extracted according to authors specified definitions and thresholds, with no additional reclassification applied."*

3. The use of proportion satisfied is a reasonable outcome measure for the meta-analysis. However, the methods used to compute study-level confidence intervals should match that used for the overall proportion. Clopper-Pearson, which is known to be overly conservative, is used for the study-level estimates, and it is unclear what is used for the overall summary. The authors only state that they have been "pooled" using a random effects meta-analysis. Is it a weighted average using the reciprocal of study level variances plus the variance of the random effect? That approach could be considered a "typical" meta-analysis approach, but Clopper-Pearson does not provide an estimate of variance for its estimate. So, the study-level information contained in the meta-analysis is not the same information that is reported at the study-level.

RESPONSE: We thank Reviewer 3 for highlighting this point. We have addressed this clarification in our response to Reviewer 2 under point 4 and trust the revisions sufficiently address the concern raised.

4. Next, it is not clear how the random effect was estimated in the meta-analysis. Was it maximum likelihood, restricted maximum likelihood, DerSimonian and Laird, or some other method?

RESPONSE: We thank Reviewer 3 for highlighting this point. We have addressed this clarification in our response to Reviewer 2, under point 4.

5. How was heterogeneity tested? The I^2 statistic does not correspond to a hypothesis test, and so that is not where the p-value comes from. Is the p-value reported from Cochran's Q statistic? If so, this should be mentioned in the methods (along with more info about the meta-analysis in general).

RESPONSE: We thank Reviewer 3 for highlighting this omission. In our analysis, we used both Cochrans Q statistic and the I^2 statistic to assess heterogeneity.

We have clarified this in two sections of the revised manuscript:

In the Data Extraction and Synthesis of the Abstract on pages 3 to 4. lines 68 to 70, we have also updated the text to reflect the use of both heterogeneity measures:

> "……*with heterogeneity evaluated using Cochran's Q statistic and I^2 and pooled estimates calculated with 95% confidence intervals (CI) using the Wilson (score) method.*

In the 'Statistical analysis' section of the Methods, on page 10, lines 218 to 221:

"Cochran's Q statistic and I^2 were used for heterogeneity and a pooled estimate with 95% confidence intervals (CI) using the Wilson (score) method. A p value of <0.10 for the Cochran's Q test or I^2 > 50% indicate heterogeneity between studies."

6. Please order the studies in the forest plot in some reasonable way, e.g., by publication year.

RESPONSE: We thank Reviewer 3 for highlighting this point. In response, studies are now listed by publication year in the forest plot, Figure 2, on page 29, to improve interpretability

7. Table 2. Listing out the countries or categorising them by continent would be much more informative than having "other" categories.

RESPONSE: We thank Reviewer 3 for this helpful suggestion. In response, we have removed the "Other" category from Table 2, on page 12, and now list all countries individually.

8. The results section seems to ramble on. Would a summary table or tables make more sense here?

RESPONSE: We thank Reviewer 3 for this comment. This point was also raised by Reviewer 1, and has been addressed in our response under point 12. We trust the revisions made adequately respond to the feedback and clarify the issue raised by both reviewers.

9. Sentence spanning pages 33-34, different approaches will result in different results. So what? Perhaps explain why these results may be unrelated to the outcome being studied.

RESPONSE: We thank Reviewer 3 for this feedback. We have rephrased our text to clarify how participant responses may vary depending on the framing and context of the survey. This content of the Discussion has been updated on page 28, lines 527 to 530 as follows:

"Patient responses are influenced by context and question framing. The same individual may respond differently to a "hospital hygiene" item in a general experience survey compared to an IPC-specific survey administered during isolation, potentially altering satisfaction estimates independent of actual experience."

10. Conclusion. I cannot say I agree that service improvement is "badly" needed. Sure, there is room for improvement with 69% satisfaction, but I would not categorise that level as badly needing improvement.

RESPONSE: We thank Reviewer 3 for this comment. This point was also raised by both Reviewer 1, and Reviewer 2. We trust we have adequately addressed this feedback and clarified the issue raised by the reviewers in our response Reviewer 1, under point 20.

VERSION 2 - REVIEW

**Reviewer**	**2**
**Name**	**Markwart, Robby**

Affiliation **Jena University Hospital, Institute of General Practice and Family Medicine**

Date **22-Sep-2025**

COI

The authors revised the manuscript to my satisfaction.

Reviewer **3**

Name **Olivier, Jake**

Affiliation **University of New South Wales, School of Mathematics and Statistics**

Date **10-Oct-2025**

COI

Thank you for addressing most of my earlier comments; however, my third comment was not addressed. I have repeated that comment below:

"The use of proportion satisfied is a reasonable outcome measure for the meta-analysis. However, the methods used to compute study-level confidence intervals should match that used for the overall proportion. Clopper-Pearson, which is known to be overly conservative, is used for the study-level estimates, and it is unclear what is used for the overall summary. The authors only state that they have been "pooled" using a random effects meta-analysis. Is it a weighted average using the reciprocal of study level variances plus the variance of the random effect? That approach could be considered a "typical" meta-analysis approach, but Clopper-Pearson does not provide an estimate of variance for its estimate. So, the study-level information contained in the meta-analysis is not the same information that is reported at the study-level."

The primary issue is that there is no connection between the study-level estimates and the overall estimate. The stated statistical methods and the forest plot give the impression the study-level data were "pooled" to get the overall estimate, which is not true. Because of this disconnect, the use of study "weights" in the forest plot is misleading, I believe.

Additionally, the revised paper states that both the Wilson (score) and the Clopper-Pearson confidence intervals were used for study-level estimates. These methods are not the same, so I am assuming there has been a mistake in the writing.

It is unclear to me why the authors have arcsine transformed the study proportions. Proportions are approximately normally distributed except for proportions near 0 or 1 ("closeness" is a function of sample size). Given that the observed proportions are in the range (0.6, 0.7), I doubt the normal assumption would be violated.

DerSimonian-Laird is probably OK for this example, but ReML is likely an overall better choice as it deals with potential bias in estimation.

VERSION 2 - AUTHOR RESPONSE

Reviewer: 2

Dr. Robby Markwart, Jena University Hospital
Comments to the Author:
The authors revised the manuscript to my satisfaction.
RESPONSE: We thank the reviewer for their input and feedback on our manuscript.

Reviewer: 3

Dr. Jake Olivier, University of New South Wales
Comments to the Author:
Thank you for addressing most of my earlier comments; however, my third comment was not addressed. I have repeated that comment below:

"The use of proportion satisfied is a reasonable outcome measure for the meta-analysis. However, the methods used to compute study-level confidence intervals should match that used for the overall proportion. Clopper-Pearson, which is known to be overly conservative, is used for the study-level estimates, and it is unclear what is used for the overall summary. The authors only state that they have been "pooled" using a random effects meta-analysis. Is it a weighted average using the reciprocal of study level variances plus the variance of the random effect? That approach could be considered a "typical" meta-analysis approach, but Clopper-Pearson does not provide an estimate of variance for its estimate. So, the study-level information contained in the meta-analysis is not the same information that is reported at the study-level."

The primary issue is that there is no connection between the study-level estimates and the overall estimate. The stated statistical methods and the forest plot give the impression the study-level data were "pooled" to get the overall estimate, which is not true. Because of this disconnect, the use of study "weights" in the forest plot is misleading, I believe.

Additionally, the revised paper states that both the Wilson (score) and the Clopper-Pearson confidence intervals were used for study-level estimates. These methods are not the same, so I am assuming there has been a mistake in the writing.

It is unclear to me why the authors have arcsine transformed the study proportions. Proportions are approximately normally distributed except for proportions near 0 or 1 ("closeness" is a function of sample size). Given that the observed proportions are in the range (0.6, 0.7), I doubt the normal assumption would be violated.

DerSimonian-Laird is probably OK for this example, but ReML is likely an overall better choice as it deals with potential bias in estimation.

RESPONSE: We thank reviewer 3 for the clarification on the use of study level and overall summary confidence intervals. We apologise for the oversight in this regard and have updated the methods and the forest plot to reflect consistent use of 95% confidence intervals in study-level and overall summary estimate. We have also updated the analysis using untransformed proportions, without the use of the arcsine transformation, as the reviewer correctly points out that the proportions are not close to 0 or 1, and we have used ReML in the random effects model as suggested by the reviewer. The 'Statistical analysis' of the 'Methods' section on page 10, lines 200 to 208 now read as:

> *"Proportions were pooled using Stata's meta routine, using untransformed proportions [24], and random effects model applied using Restricted Maximum Likelihood (ReML). Cochran's Q statistic and I^2 were used for heterogeneity. A p value of <0.10 for the Cochran's Q test or I^2 > 50% indicate heterogeneity between studies[25] All analyses were performed using Stata version 18.5.*

Reference 24 and Figure 2 (below) have both also been updated accordingly.

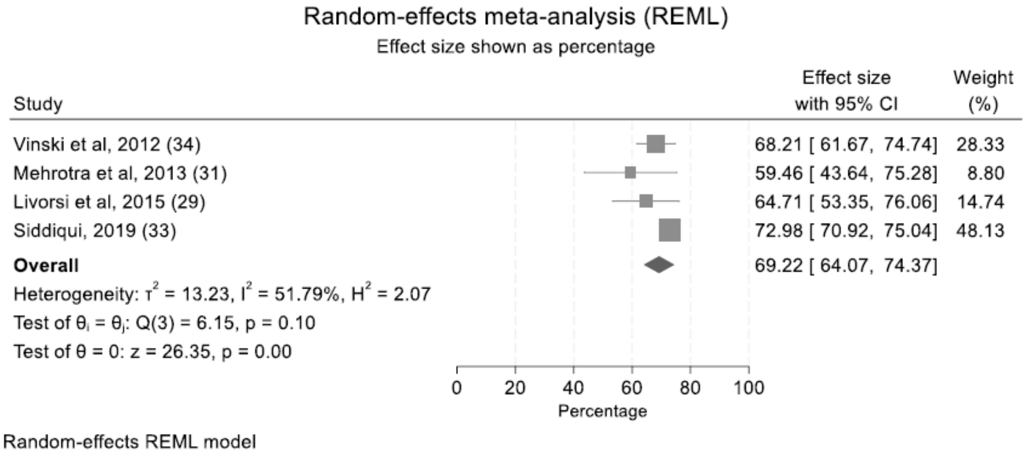

Figure 2: Forest plot of proportions of satisfied patients undergoing isolation precautions. Each row represents one study in the meta-analysis, with proportion of satisfied patients under isolation precautions (box) plotted with 95% confidence interval.(CI)

VERSION 3 - REVIEW

**Reviewer**	**3**
**Name**	**Olivier, Jake**
**Affiliation**	**University of New South Wales, School of Mathematics and Statistics**
**Date**	**12-Nov-2025**

COI

I have no further comments
